# Updated Asian consensus guidelines for the diagnosis and management of gastrointestinal stromal tumor (2025)

**DOI:** 10.1007/s10120-026-01773-4

**Published:** 2026-07-15

**Authors:** Hidekazu Hirano, Yoichi Naito, Tsuyoshi Takahashi, Seiichi Hirota, Kyoung-Mee Kim, Min-Hee Ryu, Jian Wang, Hui Cao, Jian Li, Chien-Feng Li, Chueh-Chuan Yen, Han-Kwang Yang, Hwoon-Yong Jung, Chun-Nan Yeh, Hsiu-Po Wang, Li-Tzong Chen, Lin Shen, Yoon-Koo Kang, Toshirou Nishida

**Affiliations:** 1 Department of Gastrointestinal Medical Oncology, National Cancer Center Hospital, Tokyo, Japan; 2 Department of General Internal Medicine/Experimental Therapeutics/Medical Oncology, National Cancer Center Hospital East, Chiba, Japan; 3 Department of Gastroenterological Surgery, Osaka University Graduate School of Medicine, Osaka, Japan; 4 Department of Diagnostic Pathology, School of Medicine, Hyogo Medical University, Nishinomiya, Japan; 5 Department of Pathology and Translational Genomics, Samsung Medical Center, Sungkyunkwan University School of Medicine, Seoul, Republic of Korea; 6 Department of Oncology, Asan Medical Center, University of Ulsan College of Medicine, Seoul, Republic of Korea; 7 Department of Pathology, Fudan University Shanghai Cancer Center, Shanghai, China; 8 Department of Gastrointestinal Surgery, Renji Hospital, Shanghai Jiao Tong University School of Medicine, Shanghai, China; 9 State Key Laboratory of Holistic Integrative Management of Gastrointestinal Cancers, Beijing Key Laboratory of Cell & Gene Therapy for Solid Tumor, Department of Gastrointestinal Oncology, Peking University Cancer Hospital & Institute, Beijing, China; 10 National Institute of Cancer Research, National Health Research Institutes, Tainan, Taiwan; 11 Division of Clinical Research, Department of Medical Research, and Division of Medical Oncology, Center for Immuno-oncology, Department of Oncology, Taipei Veterans General Hospital, Taipei, Taiwan; 12 School of Medicine, College of Medicine, and Institute of Biopharmaceutical Sciences, College of Pharmaceutical Sciences, National Yang Ming Chiao Tung University, Taipei, Taiwan; 13 Department of Surgery, Seoul National University Hospital, Seoul, Republic of Korea; 14 Department of Gastroenterology, Asan Medical Center, University of Ulsan College of Medicine, Seoul, Republic of Korea; 15 Department of Surgery, Chang Gung Memorial Hospital, Linkou and Chang Gung University, Taipei, Taiwan; 16 Department of Internal Medicine, National Taiwan University Hospital, National Taiwan University College of Medicine, Taipei, Taiwan; 17 Department of Internal Medicine, Kaohsiung Medical University Hospital, and College of Medicine and Center for Cancer Research, Kaohsiung Medical University, Kaohsiung, Taiwan; 18 Department of Oncology, Uijeongbu Eulji Medical Center, Eulji University, Uijeongbu-si, Republic of Korea; 19 Department of Surgery, Japan Community Healthcare Organization Osaka Hospital, 4-2-78, Fukushima, Fukushima-ku, Osaka, 553-0003 Japan; 20 Rare Cancer Center, National Cancer Center, Tokyo, Japan

**Keywords:** GIST, Consensus, Guideline, Asia, Diagnosis, Treatment

## Abstract

Gastrointestinal stromal tumor (GIST) is the most common mesenchymal tumor arising from the gastrointestinal tract. Accurate pathological diagnosis and appropriate treatment for this malignancy require a multidisciplinary approach. In consideration of the differences in clinical practice between Asian and Western countries, the Asian Consensus Guidelines for the Diagnosis and Management of GISTs were published in 2016 by multidisciplinary experts in Asian countries (Japan, Korea, China, and Taiwan). Given the accumulation of new evidence since the previous publication, a multidisciplinary expert panel consisting of pathologists, surgical oncologists, and medical oncologists revised the Asian consensus guidelines. This narrative review provides updated consensus recommendations reflecting available evidence, expert opinion, and current clinical practice for the diagnosis and management of GIST in Asian countries.

**Supplementary information:**

The online version contains supplementary material available at https://doi.org/10.1007/s10120-026-01773-4.

## Introduction

Gastrointestinal stromal tumor (GIST) is a rare type of mesenchymal tumor arising from the gastrointestinal (GI) tract with an annual incidence of approximately 1–2 per 100,000 population [1]. GIST is characterized by heterogeneity of clinicopathological and molecular features. Activating mutations in *KIT* or *PDGFRA* (plateletderived growth factor receptor alpha) are the most common driving genetic alterations for the development of GIST. The treatment paradigm for tumors with *KIT*/*PDGFRA* mutations has been revolutionized by molecular targeted agents, leading to improved survival. Multidisciplinary collaboration of pathologists, surgeons, and medical oncologists is essential for delivering appropriate and individualized care.

Previously, we published the Asian consensus guidelines based on a thorough review of the relevant literature and the diagnostic and management experience of Asian GIST experts, with the aim of providing an optimal approach for Asian patients with GIST [2]. Given the accumulation of new evidence since the previous publication, it is recommended that the guidelines be revised to reflect the current state of knowledge. Each subgroup of the Asian GIST consensus panel, consisting of experts in pathology, surgical oncology, medical oncology, and multidisciplinary treatment, was tasked with discussing and preparing updated recommendations tailored to their respective areas of expertise.

We aimed to provide updated Asian guidelines for the management of GIST.

## Methods

The current guidelines provide a structured narrative overview of key considerations in the diagnosis and treatment of GIST. We assessed the available evidence narratively, taking into account the study design, the quality and consistency of the data, clinical relevance, and applicability to Asian GIST patients. In preparing these guidelines, clinical questions (CQs) were formulated in four domains: pathological diagnosis, surgical treatment, medical therapy, and multidisciplinary treatment. To establish consensus based on scientific and clinical validity, the status of regulatory approval and insurance coverage for testing and treatment was not considered in the preparation of these guidelines. A total of 19 panel members from each country were assigned to one of the four subgroup domains, where they drafted the CQs and recommendation statements and prepared a draft for each domain (Table 1). Subsequently, the CQs, recommendation statements, and draft modifications were discussed at a web meeting attended by all panel members.

**Table 1:** Clinical questions and recommendations

				Voting results (N = 19)^a^				Consensus rate^b^
Clinical question (CQ)		Recommendation		Agree	Disagree	Neither	Abstain	
1–1	Are mutational analyses recommended in clinical practice for GIST?	1-1-1	Mutational analyses, including NGS techniques, are recommended when the diagnosis of GIST is indeterminate based on morphological and immunohistochemical findings.	19(100%)	0(0%)	0(0%)	0(0%)	100% (19/19)
		1-1-2	Mutational analyses, including NGS techniques, are recommended for advanced or metastatic GIST as well as in the neoadjuvant or adjuvant setting.	19(100%)	0(0%)	0(0%)	0(0%)	100% (19/19)
2–1	Is a biopsy necessary for the diagnosis of GIST before initiating preoperative or systemic therapy?	2-1-1	A biopsy is recommended to confirm the diagnosis of primary GIST before initiating preoperative therapy, particularly in cases where neoadjuvant or systemic treatment is being considered.	19(100%)	0(0%)	0(0%)	0(0%)	100% (19/19)
		2-1-2	Tissue sampling using endoscopic ultrasound-guided fine-needle aspiration (EUS-FNA) or fine-needle biopsy (EUS-FNB) is preferred for its safety and effectiveness in minimizing the risk of tumor hemorrhage or peritoneal dissemination.	18(94.7%)	0(0%)	0(0%)	1(5.3%)	100% (18/18)
		2-1-3	Percutaneous image-guided biopsy is generally not recommended for primary localized tumors but may be acceptable for metastatic or recurrent disease, or when EUS-FNA/FNB is not feasible in locally advanced cases.	19(100%)	0(0%)	0(0%)	0(0%)	100% (19/19)
2–2	Is laparoscopic (assisted) surgery recommended for the resection of GIST?	2-2-1	Laparoscopic surgery is recommended for smaller GIST in favorable anatomical locations, provided that standard surgical principles—such as preserving the pseudocapsule and avoiding tumor rupture—are strictly followed.	19(100%)	0(0%)	0(0%)	0(0%)	100% (19/19)
		2-2-2	For larger GIST, laparoscopic surgery may be considered in select cases, but its application should be individualized based on tumor size, location, and growth pattern.	19(100%)	0(0%)	0(0%)	0(0%)	100% (19/19)
2–3	Is tumor rupture associated with poor prognosis in patients with GIST?	2-3-1	Tumor rupture, whether occurring preoperatively or intraoperatively, is strongly associated with poor prognosis in patients with GIST, including an increased risk of recurrence.	19(100%)	0(0%)	0(0%)	0(0%)	100% (19/19)
		2-3-2	To minimize the risk of tumor rupture, careful surgical techniques and thorough preoperative planning are essential, particularly for large or high-risk tumors.	19(100%)	0(0%)	0(0%)	0(0%)	100% (19/19)
2–4	Is routine postoperative follow-up useful for completely resected GIST?	2-4-1	Routine postoperative follow-up is weakly recommended for patients with completely resected GIST.	14(73.7%)	5(26.3%)	0(0%)	0(0%)	73.7% (14/19)
		2-4-2	Follow-up schedules should be based on the risk of recurrence, time elapsed since surgery, and use of adjuvant therapy.	19(100%)	0(0%)	0(0%)	0(0%)	100% (19/19)
3–1	What is recommended after imatinib, sunitinib, and regorafenib?	3-1-1	Ripretinib is strongly recommended.	16(84.2%)	0(0%)	0(0%)	3(15.8%)	100% (16/16)
		3-1-2	Pimitespib is recommended.	12(63.2%)	0(0%)	4(21.1%)	3(15.8%)	75.0% (12/16)
3–2	What is recommended for GIST without *KIT* mutations?	3-2-1	*PDGFRA* exon 18 mutations including D842V: avapritinib is recommended.	18(94.7%)	0(0%)	0(0%)	1(5.3%)	100% (18/18)
		3-2-2	*NTRK1/3* fusions: entrectinib and larotrectinib are recommended.	17(89.5%)	0(0%)	1(5.3%)	1(5.3%)	94.4% (17/18)
		3-2-3	*BRAF* V600E mutations: dabrafenib plus trametinib is recommended.	17(89.5%)	0(0%)	0(0%)	2(10.5%)	100% (17/17)
3–3	Are higher doses of imatinib recommended?	3-3-1	Dose escalation of imatinib is recommended for GIST with *KIT* exon 9 mutations.	16(84.2%)	2(10.5%)	0(0%)	1(5.3%)	88.9% (16/18)
3–4	Is starting with a lower dose of multikinase inhibitor acceptable?	3-4-1	Starting at a lower dose may be considered for patients who have comorbidities or who are unlikely or deemed unable to tolerate the standard dose.	17(89.5%)	0(0%)	0(0%)	2(10.5%)	100% (17/17)
4–1	What is the optimal duration of adjuvant imatinib therapy for high-risk GIST after radical resection?	4-1-1	Adjuvant imatinib therapy for 3 years is strongly recommended.	18(94.7%)	0(0%)	0(0%)	1(5.3%)	100% (18/18)
		4-1-2	Adjuvant imatinib therapy for 6 years can be recommended.	12(63.2%)	5(26.3%)	1(5.3%)	1(5.3%)	66.7% (12/18)
		4-1-3	Extended or lifelong adjuvant imatinib therapy is recommended for ruptured GIST.	16(84.2%)	0(0%)	2(10.5%)	1(5.3%)	88.9% (16/18)
4–2	Is imatinib useful as neoadjuvant therapy for large GIST?	4-2-1	Neoadjuvant imatinib therapy is recommended for large GIST (e.g., > 10 cm in diameter), GIST for which curative resection is considered highly unlikely, and GIST located in a specific area where multivisceral resection is needed.	17(89.5%)	1(5.3%)	0(0%)	1(5.3%)	94.4% (17/18)
4–3	Is surgical resection useful as an initial treatment for metastatic or recurrent GIST?	4-3-1	Surgical resection as an initial treatment is not recommended for metastatic or recurrent GIST in general.	16(84.2%)	1(5.3%)	1(5.3%)	1(5.3%)	88.9% (16/18)
		4-3-2	Surgical resection can be adopted as initial treatment for metastatic or recurrent GIST requiring local tumor control and for metastatic or recurrent wild-type GIST for which no effective systemic treatment is available.	17(89.5%)	1(5.3%)	0(0%)	1(5.3%)	94.4% (17/18)
4–4	Is surgical resection useful for metastatic or recurrent GIST that respond to drug therapy?	4-4-1	Surgical resection is not routinely recommended for metastatic or recurrent GIST that respond to imatinib.	14(73.7%)	1(5.3%)	2(10.5%)	2(10.5%)	82.4% (14/17)
		4-4-2	Given the lack of definitive evidence, the indications of surgical resection for advanced GIST during drug therapy should be individualized and discussed by a multidisciplinary team of experts.	18(94.7%)	0(0%)	0(0%)	1(5.3%)	100% (18/18)

a: Voting results may not sum to 100.0% due to rounding. b: Consensus rate was based on valid responses from 19 members.

*BRAF*, B-Raf proto-oncogene, serine/threonine kinase; EUS-FNA, endoscopic ultrasound-guided fine-needle aspiration; EUS-FNB, endoscopic ultrasound-guided fine-needle biopsy; GIST, gastrointestinal stromal tumor; *KIT*, KIT proto-oncogene, receptor tyrosine kinase; NGS, next-generation sequencing; *NTRK*, neurotrophic tyrosine receptor kinase; *PDGFRA*, platelet-derived growth factor receptor alpha

The distribution of opinions among the panel members was assessed by voting, which was done anonymously to ensure the independence of individual judgements. Responses of “Agree,” “Disagree,” and “Neither” were included in the denominator, whereas responses of “Abstain’” were excluded. As these consensus guidelines were developed through a narrative review rather than a systematic review, formal grading of the level of evidence using a structured system such as GRADE was not performed. The consensus rate was not predefined as a criterion for determining consensus strength.

## Results

### Pathological diagnosis

#### Overview of the clinicopathological features

Although GIST can arise in any portion of the GI tract, it usually occurs in the stomach (60%) or small intestine (30%). Less frequently, it involves the rectum (5%), esophagus (1%), colon (< 1%), or appendix (< 1%). GIST rarely occurs in extra-GI locations, such as the omentum, mesentery, pelvis, and retroperitoneum; however, most of these cases may actually be derived from the gastric, duodenal, intestinal, or rectal wall [3, 4]. GISTs range in size from a few millimeters in diameter to huge lesions. Smaller GISTs are often discovered incidentally during esophagogastroduodenoscopy, which is often performed at regular medical check-ups, especially in Asian countries. Detailed histopathological examinations of resected specimens for gastric cancer may also reveal coexisting minute gastric GIST [5].

In the macroscopic cross-section, GISTs typically appear as a well-circumscribed whitish, translucent mass. Large tumors often exhibit hemorrhage, necrosis, and cystic changes. GISTs can be histologically classified into three subgroups: the spindle cell type (70%), characterized by cells with ovoid to spindle nuclei; the epithelioid cell type (20%), composed of round or polygonal cells arranged in sheets and nests; and the mixed cell type (10%), which features a combination of spindle and epithelioid cells.

GISTs have various oncogenic mutations in *KIT*, *PDGFRA*, and succinate dehydrogenase (SDH) complex genes (*SDHs*), among others. GISTs with specific mutations often exhibit characteristic morphological features. Unlike typical GISTs, some GISTs—particularly recurrent tumors following imatinib therapy—may have dedifferentiated pleomorphic sarcoma-like morphology including the presence of multinucleated giant cells.

#### Immunohistochemical staining

Immunohistochemistry (IHC) is recommended to confirm the diagnosis of GIST, including cases that are suspected or even definitively identified through histological examination using hematoxylin–eosin staining. Because the initial pathological diagnosis of GIST is based on the histological morphology of the tumor, tumors lacking the typical morphological features of GIST cannot be easily diagnosed as GIST, even if some characteristic markers for GIST are positive. The most important positive IHC markers for the diagnosis of GIST are c-KIT (CD117) and DOG-1 (discovered on GIST). Approximately 95% of GISTs are diffusely and strongly positive for c-KIT, while other GI mesenchymal tumors (e.g., GI Ewing sarcoma, leiomyosarcoma, and perivascular epithelioid cell tumor [PEComa]) may be negative or partially and weakly positive. DOG-1 is positive in 95% of c-KIT-positive GISTs and in one-third of c-KIT-negative GISTs [6]. CD34 is positive in 60–80% of all GISTs, with over 90% of gastric GISTs showing positivity. However, it should be noted that other types of neoplasms that develop in the GI tract, such as inflammatory fibroid polyp and solitary fibrous tumors, can also be positive for CD34. SDHB IHC is used to diagnose SDH-deficient GIST. This type of GIST is observed in Carney-Stratakis syndrome [7] and the Carney triad [8], but sporadic SDH-deficient GIST is rare.

**Fig. 1:**
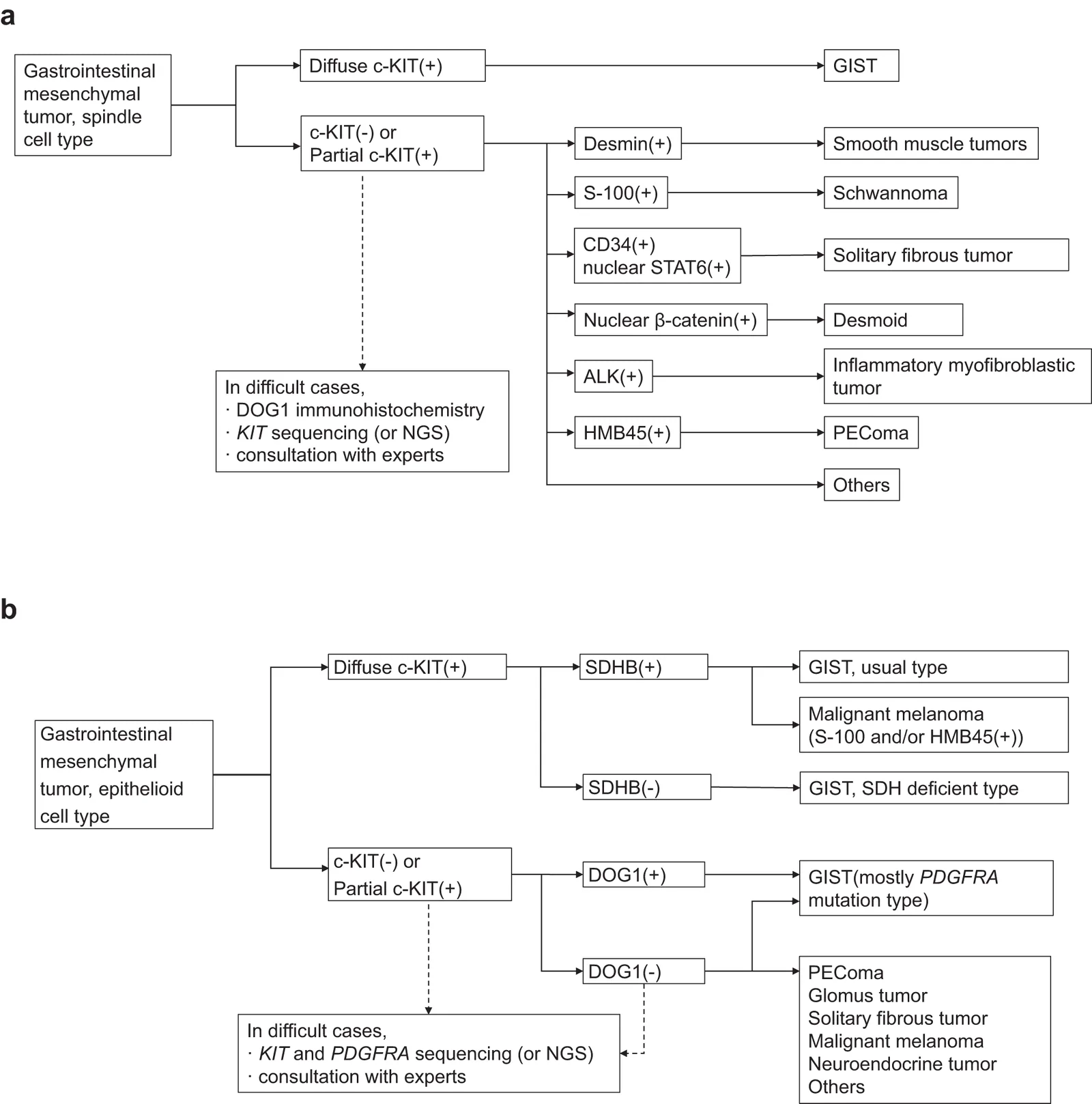
Diagnostic algorithm for gastrointestinal mesenchymal tumors. **a** Spindle cell type. ALK, anaplastic lymphoma kinase; CD34, cluster of differentiation 34; DOG1, discovered on GIST-1; GIST, gastrointestinal stromal tumor; HMB45, human melanoma black 45; KIT (c-KIT), KIT proto-oncogene, receptor tyrosine kinase; NGS, next-generation sequencing; PEComa, perivascular epithelioid cell tumor; S-100, S-100 protein; STAT6, signal transducer and activator of transcription 6. **b** Epithelioid cell type. DOG1, discovered on GIST-1; GIST, gastrointestinal stromal tumor; HMB45, human melanoma black 45; KIT (c-KIT), KIT proto-oncogene, receptor tyrosine kinase; NGS, next-generation sequencing; *PDGFRA*, platelet-derived growth factor receptor alpha; PEComa, perivascular epithelioid cell tumor; SDHB, succinate dehydrogenase subunit B; S-100, S-100 protein

The algorithms for diagnosing GIST using IHC are provided in Figs. 1a and b, categorized by tumor cell morphology. Spindle-type GISTs are almost always diffusely and strongly positive for c-KIT; therefore, spindle-type GI mesenchymal tumors that are negative or partially and weakly positive for c-KIT are not considered GISTs. For epithelioid mesenchymal tumors, a diagnosis of GIST can be made if c-KIT IHC is diffusely and strongly positive. In cases of epithelioid mesenchymal tumors that are negative or weakly positive for c-KIT, a positive DOG1 IHC result may support a diagnosis of GIST. However, it is important to note that GI melanomas—though not mesenchymal tumors—can also exhibit strong and diffuse KIT positivity. Since c-KIT-negative GISTs almost always harbor *PDGFRA* mutations, the detection of *PDGFRA* mutation may confirm the diagnosis of GIST. However, it should be noted that most inflammatory fibroid polyps also carry *PDGFRA* mutations. For the appropriate differential diagnosis of GI mesenchymal tumors other than GIST, IHC markers such as CD34, smooth muscle actin (SMA), desmin, S-100, signal transducer and activator of transcription 6 (STAT6), beta-catenin and transcription factor E3 (TFE3) can be utilized. For detailed immunohistochemical examination protocols specific to other tumor types, please refer to the relevant textbooks.

#### Various mutations in GIST

Primary *KIT* mutations, present in 80–85% of all GISTs, are most frequently observed at exon 11 (65–70%). Mutations in exon 9 are found in approximately 10% of GISTs, while those in exons 13/14 and 17/18 are rare, and those in exon 8 are extremely rare. The prognosis is considered worse for GISTs with deletion and deletion-insertion type mutations at exon 11, particularly those involving codons 557 and 558, than for GISTs with duplication or substitution mutations at exon 11 [9]. Although exon 11 mutations can occur in GISTs throughout the GI tract, exon 9 mutations are mostly found in GISTs located in the duodenum, small intestine, or rectum. Most GISTs with *KIT* mutations exhibit spindle cell morphology, though some of them may have epithelioid features. In contrast, GISTs with *PDGFRA* mutations, which occur in 5–10% of all GISTs, are typically found in exons 12, 14, or 18. These mutations are almost exclusively observed in the stomach and have an epithelioid morphology [10]. While many *PDGFRA-*mutated GISTs show clinically indolent behavior, those with D842V mutation in exon 18—the most common *PDGFRA* mutation—show resistance to imatinib.

Although proliferation of most GISTs is driven by gain-of-function mutations of *KIT* or *PDGFRA*, a small subset (10–15%) lacks these mutations. This type of GIST may harbor mutations in *SDHx* [7], *NF-1* [11] or *BRAF* [12]. Germline mutations in *SDHx* may cause Carney-Stratakis syndrome [7]. Germline mutations in *NF-1* may cause neurofibromatosis type 1 (NF1) syndrome, or von Recklinghausen syndrome, which is frequently associated with multiple small intestinal GISTs [11]. *BRAF-*mutated GISTs with V600E at exon 15 almost always have spindle cell morphology and predominantly develop in the small intestine [12]. *KRAS* or *NRAS* mutations are considered to be extremely rare if present at all [13]. Additionally, the detection of some fusion genes including *NTRK3* and *FGFR1* fusions has been reported in rare cases of GIST [14, 15]. However, it remains controversial whether GIST-like tumors with *NTRK* fusions in young children represent true GIST [16, 17].

**Fig. 2:**
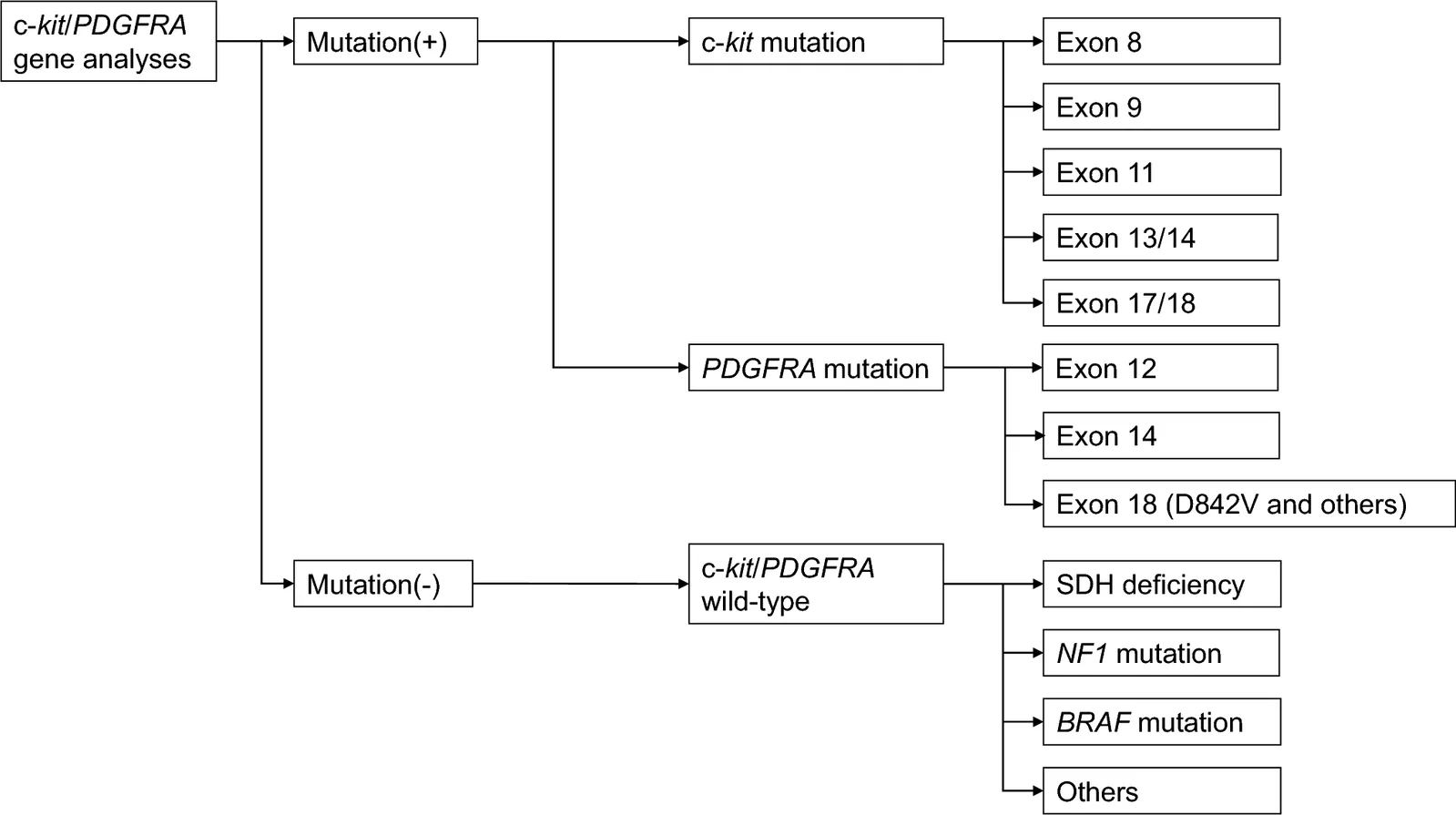
Genetic classification of GISTs. *BRAF*, B-Raf proto-oncogene, serine/threonine kinase; KIT (c-KIT), KIT proto-oncogene, receptor tyrosine kinase; NF1, neurofibromin 1; *PDGFRA*, platelet-derived growth factor receptor alpha; SDH, succinate dehydrogenase

Individual mutational analyses for *KIT* exons 8, 9, 11, 13/14, and 17/18, as well as for *PDGFRA* exons 12, 14, and 18, can be performed using both formalin-fixed, paraffin-embedded tissue and fresh-frozen tissue specimens. The algorithm for mutational analyses in GIST is shown in Fig. 2. Comprehensive mutational analyses using next-generation sequencing methods can be applied under several conditions. Additionally, mutational analyses of genes specific to other types of mesenchymal tumors may aid in the differential diagnosis of GIST. Mutational analyses are recommended when the diagnosis of GIST is not determined based on histopathological findings and when the tyrosine kinase inhibitors are used (Table 1; see CQ1-1 in the Supplementary Material for details).

#### Risk classification for localized GIST

Evaluation of the risk of recurrence after curative surgery for localized GIST can provide information about the need for adjuvant imatinib therapy. The National Institutes of Health (NIH) consensus criteria published in 2002 included two prognostic factors: tumor size and mitotic index [18]. In contrast, the Armed Forces Institute of Pathology (AFIP) criteria expanded this framework by incorporating tumor location alongside size and mitotic count [19] (Table 2). Furthermore, the modified NIH classification proposed by Joensuu [20, 21] added rupture as an additional prognostic factor (Table 3). Prognostic contour maps generated through the pooled data of worldwide GIST patients who did not receive adjuvant therapy are also used to estimate the 10-year recurrence rate for each patient [22]. These maps are categorized by the primary site and the presence of tumor rupture, with each map including the mitotic index and tumor size as continuous variables. While the optimal classification for selecting candidates for adjuvant therapy remains unclear, both the AFIP criteria and the modified NIH criteria may be recommended. The AFIP criteria are widely used in Western countries. A report from Japanese researchers indicated that the modified NIH criteria are the most sensitive for detecting patients at high risk of recurrence [23]. Additionally, a report from a Korean group highlighted the prognostic significance of tumor rupture, even in patients who received 3 years of adjuvant imatinib therapy [24]. Tumor rupture must be appropriately evaluated according to the reported proposal.

**Table 2:** Representative risk classifications. AFIP classification

Tumor parameters		Recurrence risk classification			
Tumor size (cm)	Mitotic counts (/50 HPF)^a^	Stomach	Small intestine	Duodenum	Rectum
≤ 2	≤ 5	None	None	None	None
> 2 to ≤ 5	≤ 5	Very low	Low	Low	Low
> 5 to ≤ 10	≤ 5	Low	Moderate	High	High
> 10	≤ 5	Moderate	High	High	High
≤ 2	> 5	None	High	No data	High
> 2 to ≤ 5	> 5	Moderate	High	High	High
> 5 to ≤ 10	> 5	High	High	High	High
> 10	> 5	High	High	High	High

a: “50 HPF” is “equivalent to 5 mm^2^ [19]

HPFs, high-power fields

**Table 3:** Representative risk classifications. Modified NIH classification

Tumor parameters		Recurrence risk classification	
Tumor size (cm)	Mitotic count (/50 HPF)^a^	Stomach	Other sites
< 2	≤ 5	Very low	Very low
> 2 to ≤ 5	≤ 5	Low	Low
> 5 to ≤ 10	≤ 5	Intermediate	High
≤ 2	> 5 to ≤ 10	Intermediate	High
> 2 to ≤ 5	> 5 to ≤ 10	Intermediate	High
> 5 to ≤ 10	> 5 to ≤ 10	High	High
Tumor size > 10 cm (any mitotic count)		High	High
Mitotic count > 10/50 HPF (any tumor size)		High	High
Presence of tumor rupture (any mitotic count and/or tumor size)		High	High

a: “50 HPF” is not clearly defined in the original articles [20, 21]. The guidelines define “50 HPF” as equivalent to 5 mm^2^

HPF, high-power field

#### Syndromic GIST

Multiple GISTs may develop in families with particular germline mutations. First, germline mutations of *KIT* cause multiple GISTs in the stomach and small intestine. The mutations are present in exon 8, 9, 11, 13, or 17 [25]. Diffuse hyperplasia of the interstitial cells of Cajal (ICC), which is regarded as a precancerous lesion associated with the development of GIST, is almost always observed as a characteristic finding in affected patients. Patients of these families may have mastocytoma and/or pigmentation on the hands, perioral area and/or perineum, and some may complain of dysphagia caused by impaired esophageal motility. To confirm the presence of germline *KIT* mutations, genomic mutational analyses using peripheral blood leukocytes or normal tissue should be performed. Germline mutations of *PDGFRA* may cause multiple GIST in the GI tract; however, most mesenchymal tumors in those patients do not appear to be GIST [26].

Second, germline mutations of *NF1* cause NF-1, and patients with NF-1 may develop multiple GIST in the GI tract, especially in the duodenum and small intestine. However, some patients with NF-1 may also present with multiple GIST in the stomach [27]. Most NF-1 associated GIST exhibits a spindle cell morphology. Patients with NF-1 also might show ICC hyperplasia-like findings, though these are typically scattered rather than diffuse. The diagnosis of NF-1-related GIST usually relies on the identification of NF-1 in patients based on characteristic symptoms and signs.

Third, germline mutations of *SDHx* including *SDHA*, *SDHB*, *SDHC*, or *SDHD* are associated with the development of epithelioid gastric GIST and paragangliomas (Carney-Stratakis syndrome) [7]. These tumors are characteristically immunohistochemically negative for SDH. A subset of SDH-deficient GISTs may arise due to epigenetic abnormalities of *SDHC* (Carney triad) [8]. Patients with the Carney triad typically present with epithelioid gastric GIST, pulmonary chondromas, or extra-adrenal paragangliomas. Although Carney-Stratakis syndrome affects both sexes equally, the Carney triad shows a female predominance. Most patients with the Carney triad have only two of the three components, and the second component often occurs after a long interval following the detection of the first component. An SDH-deficient gastric GIST occurring without associated tumors may simply be referred to as an SDH-deficient GIST; however, genetic analyses to detect *SDH* mutations and epigenetic abnormality of *SDHC* are necessary to determine the underlying condition. Both types of SDH-deficient GISTs almost always occur in the stomach, exhibit epithelioid morphological features, and show diffuse and strong KIT immunoreactivity. They often show characteristic multiple or lobulated/plexiform growth patterns in the muscularis propria of the stomach, along with frequent lymphovascular invasion and lymph node metastases [28]. Since most of those types of GISTs develop in childhood or adolescence, SDHB IHC should be performed in young patients presenting with epithelioid gastric GIST.

#### Pathologic reporting for GIST

Pathology reports for GIST should include information about the tumor location, size, mitotic index (per 5 mm^2^), resection margin status, and IHC results (e.g., c-KIT and/or DOG-1). It is also recommended to include a description of the recurrence risk grade based on the AFIP criteria or modified NIH criteria (Table 2, 3) [19–21]. Because the size of a high-power field may vary across microscopes, mitotic counts should be expressed as the number of mitotic figures per 5-mm^2^ field. Additional histological characteristics of the tumor may be included, such as the degree of cellularity, presence of necrosis, cystic degeneration, vessel invasion, and invasion into the mucosa or adjacent structures. The panel recommends documenting the results of mutational analyses conducted in centralized laboratories or medical centers with quality assurance protocols.

### Surgical treatment

#### Indication for resection of GIST

Surgery remains the cornerstone of curative treatment for GIST and represents the only modality with curative potential in patients with localized disease [29, 30]. Given the inherent malignant potential of GIST, complete surgical resection is generally recommended whenever the tumor is localized and technically resectable, regardless of tumor size or location.

For small gastric GIST measuring less than 2 cm without apparent high-risk features [31], non-operative management with active surveillance may be an acceptable option [32]. This approach may be considered particularly when endoscopic and imaging findings demonstrate regular margins, a homogeneous appearance, and no evidence of interval growth. However, there are reports of late recurrence and cancer-specific mortality even for tumors smaller than 2 cm, suggesting that these lesions cannot be regarded as entirely indolent [33]. Accordingly, surgical resection may still be considered for small gastric GIST after appropriate patient counseling, taking into account tumor characteristics, surgical risk, and patient preference.

#### Importance of biopsy-based diagnosis before treatment

Histological diagnosis is generally required to confirm primary GIST before treatment planning, and is particularly important when preoperative or systemic therapy, including neoadjuvant treatment, is being considered (Table 1; see CQ2-1 in the Supplementary Material for details). Endoscopic ultrasound–guided fine-needle aspiration or biopsy (EUS-FNA/FNB) is preferred, as it provides sufficient diagnostic accuracy while minimizing the risk of tumor hemorrhage or peritoneal dissemination [34–36]. Compared with EUS-FNA, EUS-FNB allows larger tissue samples to be obtained and facilitates immunohistochemical and mutational analyses [37]. Alternative methods, such as mucosal incision-assisted biopsy, have also demonstrated diagnostic accuracy and safety comparable to EUS-guided techniques [38]. Percutaneous image-guided biopsy is not routinely recommended for primary localized GIST but may be acceptable in metastatic or recurrent disease or when EUS-guided sampling is not feasible. Retrospective studies suggest that, when combined with appropriate systemic therapy, percutaneous biopsy does not necessarily increase recurrence risk in high-risk GIST [39, 40]. Therefore, the biopsy strategy should be individualized, balancing diagnostic necessity, technical feasibility, and oncologic safety.

#### Principles of surgical management for GIST

The primary objective of surgery is complete gross resection with macroscopically negative margins [41]. Because GIST typically exhibits expansive rather than infiltrative growth, wide margins are unnecessary, and segmental or wedge resection is usually sufficient [41]. Routine lymphadenectomy is not indicated, as lymph node metastasis is rare in GIST. An exception is SDH–deficient GIST, which has a higher incidence of nodal involvement; even in such cases, selective removal of clinically enlarged lymph nodes is considered sufficient [42].

Minimally invasive approaches should be considered within the framework of these surgical principles. Laparoscopic surgery is recommended for smaller GISTs located in favorable anatomical sites, provided that oncologic safety—particularly preservation of the pseudocapsule and avoidance of tumor rupture—is strictly maintained (Table 1; see CQ2-2 in the Supplementary Material for details) [43–45]. Retrospective studies have shown comparable oncologic outcomes between laparoscopic and open surgery, with additional benefits for laparoscopic surgery including reduced postoperative pain, shorter hospital stays, and faster recovery [43]. For larger tumors, laparoscopic resection may be considered in carefully selected cases at experienced centers, although supporting evidence remains limited. Tumors exceeding 8 cm require particularly cautious preoperative evaluation and surgical planning [45–47]. In all laparoscopic procedures, appropriate tumor manipulation, use of stapling devices, and specimen retrieval with protective bags are essential to minimize the risk of tumor rupture [44]. Laparoscopic and endoscopic cooperative surgery has also been applied to selected gastric GISTs, offering organ-preserving resection with acceptable short-term outcomes [48, 49]. In contrast, endoscopic full-thickness resection remains controversial because of concerns regarding tumor spillage and uncertainty about long-term oncologic safety [50–53].

#### Tumor rupture

**Fig. 3:**
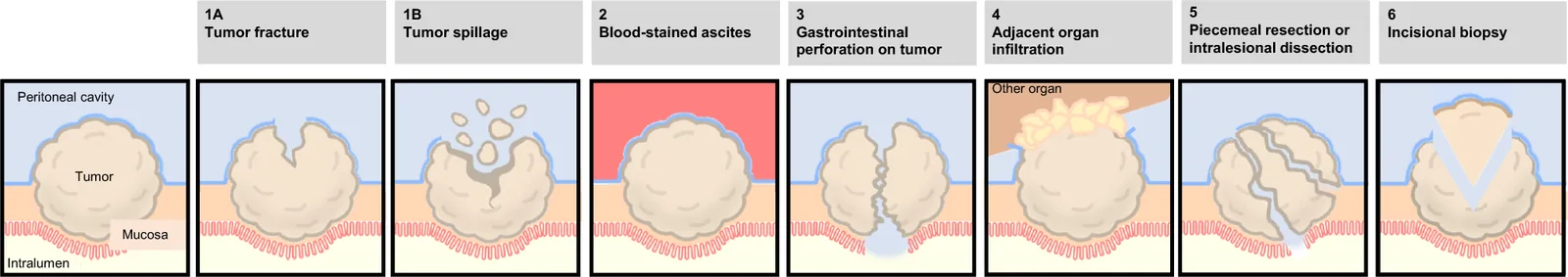
Definitions of tumor rupture for GISTs

Tumor rupture, which is presumably associated with micrometastatic disease, is one of the most important adverse prognostic factors for recurrence (Table 1; see CQ2-3 in the Supplementary Material for details) [21, 54, 55]. Rupture may occur preoperatively or intraoperatively and reflects both aggressive tumor biology and technical factors. To standardize prognostic evaluation, a unified definition of tumor rupture has been proposed, encompassing events such as tumor fracture, spillage of tumor contents, blood-stained ascites, perforation at the tumor site, adjacent organ infiltration, piecemeal resection, and incisional biopsy (Fig. 3) [56, 57]. Patients meeting this definition represent an extremely high-risk group [22] and often require prolonged or lifelong adjuvant imatinib therapy.

#### Postoperative follow-up for completely resected GIST

Postoperative surveillance is weakly recommended after complete resection of GIST, although the supporting evidence is derived primarily from expert consensus and observational studies (Table 1; see CQ2-4 in the Supplementary Material for details) [58–60]. Follow-up strategies should be individualized based on recurrence risk, time elapsed since surgery, and whether adjuvant therapy is administered. Observational studies suggest that early detection of recurrence, particularly when tumor burden is limited, may be associated with improved outcomes [61, 62]. Accordingly, patients with intermediate- or high-risk GIST typically undergo more intensive surveillance with contrast-enhanced computed tomography during the first several years after surgery, while less frequent imaging may be sufficient for low-risk patients [60, 61, 63]. The role of positron emission tomography in routine surveillance has not been established.

### Medical therapy

#### Current standard of care for advanced GIST

Metastatic or recurrent GIST is generally incurable, and the primary goals of medical therapy are to prolong survival and alleviate symptoms through systemic treatment. Most GISTs harbor activating mutations in the *KIT* gene, making them amenable to molecularly targeted therapies (Table 4). Although randomized trials comparing imatinib with supportive care are lacking, imatinib is the established first-line treatment for advanced GIST. The pivotal B2222 trial demonstrated its strong efficacy, with the objective response rate (ORR) of 68.1%, a median time to progression of 24 months, and a 5-year overall survival (OS) rate of 55% [64].

**Table 4:** Medical therapy for advanced/recurrent GISTs

GIST	First line	Second line	Third line	Fourth line	Thereafter
*KIT* mutant	Imatinib	Sunitinib	Regorafenib	Ripretinib Pimitespib	Drugs that were formerly effective
*PDGFRA* exon 18 mutant	Avapritinib	–	–	–	Drugs that were formerly effective
*NTRK1/3* fusion	Entrectinib Larotrectinib	–	–	–	Drugs that were formerly effective
*BRAF* V600E mutant	Dabrafenib + Trametinib	–	–	–	Drugs that were formerly effective

*BRAF,* v-raf murine sarcoma viral oncogene homolog B; GIST, gastrointestinal stromal tumors; *KIT*, KIT proto-oncogene, receptor tyrosine kinase; *NTRK,* neurotrophic tropomyosin receptor kinase; *PDGFRA,* platelet-derived growth factor receptor alpha. It should be noted that the availability of drugs varies across countries because of differences in regulatory approval, reimbursement status, and insurance coverage

Sunitinib is the standard second-line therapy following imatinib failure. In randomized studies, sunitinib significantly extended time to progression compared with placebo (median time to progression: 27.3 weeks vs. 6.4 weeks), although the ORR was modest, at 7% [65]. Resistance to imatinib most commonly arises from secondary *KIT* mutations, particularly in the ATP-binding domain (exons 13/14) or activation loop (exons 17/18). The activity of targeted agents varies depending on the location of these secondary mutations [66]. For example, sunitinib appears more effective in tumors with ATP-binding domain mutations, whereas newer agents such as regorafenib, ripretinib and avapritinib demonstrate greater activity against activation loop mutations [67–69].

Regorafenib is the standard third-line therapy after failure of imatinib and sunitinib, providing a modest but significant improvement in progression-free survival (PFS) compared with placebo (median PFS: 4.8 months vs. 0.9 months) after the failure of imatinib and sunitinib, though the ORR remained low, at 4.5% [70]. With advances in drug development, the therapeutic focus has increasingly shifted toward fourth-line treatment for heavily pretreated GIST.

#### Treatment options following imatinib, sunitinib, and regorafenib

For patients previously treated with imatinib, sunitinib, and regorafenib, ripretinib is strongly recommended as fourth-line therapy (Table 1; see CQ3-1 in the Supplementary Material for details). Ripretinib is a novel oral inhibitor targeting KIT and PDGFRA. In the phase 3 INVICTUS trial, ripretinib significantly improved PFS and showed a trend toward improved OS compared with placebo, with generally mild and tolerable toxicities such as alopecia, myalgia, nausea, and fatigue [71]. Pimitespib, an oral heat shock protein 90 inhibitor available in Japan, is also recommended, though with less consensus. In a phase 3 trial, pimitespib improved both PFS and OS relative to placebo, with diarrhea and appetite loss as common adverse events [72]. Although both agents are recommended, ripretinib is favored due to its greater magnitude of benefit and tolerability.

Direct comparisons between ripretinib and pimitespib are not available, and the optimal sequencing or combination of these agents remains unclear. Ongoing trials are evaluating ripretinib versus sunitinib in selected molecular subgroups [73] and pimitespib in combination with imatinib (jRCT2011210044) [74]. When newer agents are unavailable or ineffective, rechallenge with previously effective therapies, such as imatinib, may be considered, supported by evidence from the RIGHT study [75].

#### Treatment options for GIST without *KIT* mutations

Management strategies differ for GIST lacking *KIT* mutations (Table 1; see CQ3-2 in the Supplementary Material for details). GISTs with *PDGFRA* exon 18 mutations, particularly D842V, are resistant to imatinib [76] but respond well to avapritinib. The NAVIGATOR study demonstrated a high response rate in this population, leading to regulatory approval despite notable adverse events including cognitive effects and rare intracranial bleeding [77]. Wild-type GISTs, lacking both *KIT* and *PDGFRA* mutations, may harbor other actionable alterations. Tumors with *NTRK* fusions respond dramatically to TRK inhibitors such as entrectinib and larotrectinib [78–81], while those with *BRAF* V600E mutations may benefit from combined RAF and MEK inhibition using dabrafenib and trametinib [82]. In contrast, SDH-deficient GISTs [83] show limited sensitivity to imatinib but may derive some benefit from sunitinib or regorafenib [84–87]. For indolent wild-type GISTs, active surveillance is also a reasonable option.

#### Dose escalation of imatinib

Dose escalation of imatinib to 800 mg daily may be considered for patients with *KIT* exon 9 mutations, particularly after progression on the standard dose (Table 1; see CQ3-3 in the Supplementary Material for details) [88]. Evidence suggests higher response rates and improved PFS but no OS benefit, and higher doses may be less favorable than switching to sunitinib [89–92]. It should be noted that a retrospective study found no survival benefit with an imatinib dose of 800 mg compared with 400 mg in patients with *KIT* exon 9 mutated GIST in the adjuvant setting [93]. Therefore, dose escalation should be applied cautiously, especially in Asian patients.

#### Dose-reduced multikinase inhibitors

While standard dosing of multikinase inhibitors is recommended, starting at a lower dose may be appropriate for selected patients with significant comorbidities or limited tolerance (Table 1; see CQ3-4 in the Supplementary Material for details) [94]. Monitoring plasma imatinib levels can support dose adjustment decisions in selected situations [95–98]. Individualized dose optimization based on toxicity and patient factors is emphasized, as appropriate dose adjustments do not appear to compromise efficacy.

### Multidisciplinary treatment

#### Adjuvant imatinib therapy for high-risk GIST

**Fig. 4:**
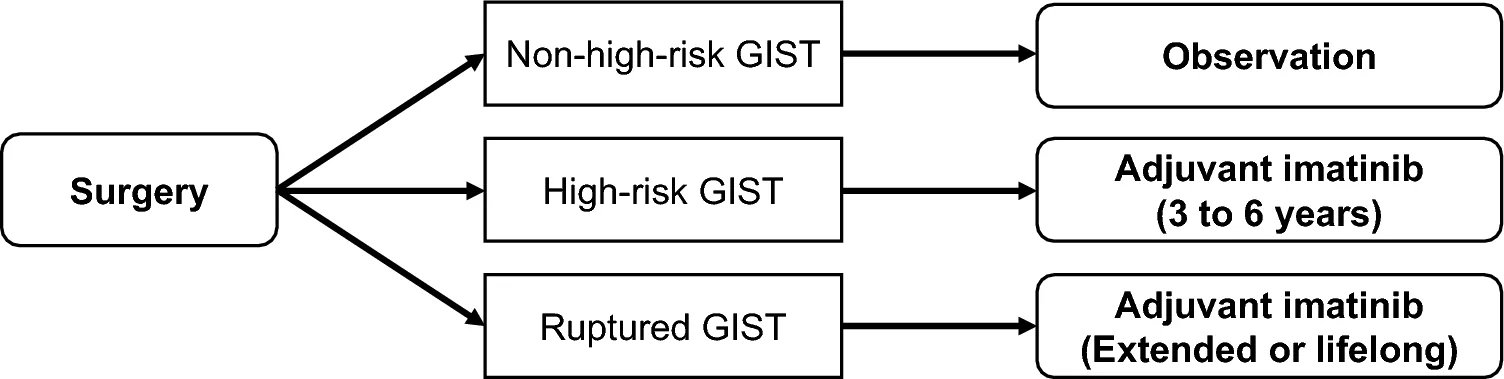
Postoperative treatment recommendations for GISTs. GIST, gastrointestinal stromal tumor

As previously discussed, the panel recommends that the modified NIH criteria be used to guide the selection of patients who may require adjuvant imatinib therapy (Fig. 4). The ACOSOG Z9001 study demonstrated that adjuvant imatinib therapy for 1-year prolonged recurrence-free survival (RFS) compared with placebo after curative resection of a primary GIST [99]. In the EORTC 62024 study, patients who received 2 years of adjuvant imatinib therapy showed a trend toward improved imatinib failure-free survival relative to the observation group [100, 101]. Subsequently, the SSG XVIII/AIO study demonstrated that adjuvant therapy with imatinib for 3 years improved RFS and OS relative to adjuvant therapy with imatinib for 1 year in patients with high-risk GIST [102, 103]. Based on the SSG XVIII/AIO study results, adjuvant therapy with imatinib for 3 years is strongly recommended for high-risk GIST after surgical resection according to the modified NIH criteria (Table 1; see CQ4-1 in the Supplementary Material for details). On the other hand, the optimal duration of imatinib adjuvant therapy remains under consideration. The IMADGIST phase 3 trial demonstrated that extending adjuvant imatinib for an additional 3 years significantly improved disease-free survival in high-risk GIST patients who had completed 3 years of treatment, though OS data remains immature [104]. Extended or lifelong adjuvant imatinib therapy is recommended for ruptured GIST, as those patients are considered to have an increased risk of recurrence. A genotype analysis of GIST patients being evaluated for adjuvant imatinib therapy is preferable for identifying imatinib-insensitive GISTs, including *PDGFRA* D842V mutant GIST and wild-type GIST.

#### Neoadjuvant imatinib therapy for localized GIST

Neoadjuvant imatinib therapy can be considered to increase the likelihood of curability as well as the safety of surgery and organ function preservation in cases where the GIST is large (e.g., > 10 cm in diameter) or difficult to radically resect without significant morbidity [105–110] (Table 1; see CQ4-2 in the Supplementary Material for details). Imatinib is the only drug with evidence to support its use in the neoadjuvant setting. Although the optimal duration of neoadjuvant imatinib therapy remains undefined, surgery is generally recommended after 6–12 months of treatment to balance maximal tumor shrinkage with the risk of secondary resistance associated with prolonged administration [64, 111]. In patients being evaluated for neoadjuvant imatinib therapy, a genotype analysis is preferable for identifying imatinib-insensitive GIST, including *PDGFRA* D842V-mutant GIST and wild-type GIST. After neoadjuvant therapy, risk classification for recurrence using surgical specimens cannot be applied due to pathological modifications by imatinib.

#### Upfront surgical resection for advanced GIST

Considering that micrometastasis or circulating tumor cells may already be present at the time of diagnosis, surgical resection is not generally recommended as the initial treatment for metastatic or recurrent GIST, and drug therapies including imatinib are the standard of care (Table 1; see CQ4-3 in the Supplementary Material for details). There are currently no established subgroups of patients with advanced GIST in whom upfront surgical resection has been shown to be beneficial. Surgical resection can be adopted as initial treatment for metastatic or recurrent GIST requiring local tumor control and for metastatic or recurrent wild-type GIST for which no effective systemic treatment is available.

#### Surgical resection following drug therapy for advanced GIST

Systemic treatment rarely eradicates all tumor cells, resulting in a low pathological complete response rate due to the existence and/or emergence of drug-resistant tumor clones. Drug-resistant tumor clones are thought to be present before treatment and/or develop over time after treatment. Given that surgical resection may play a role in eliminating or delaying the outgrowth of drug-resistant tumor clones, surgical resection can be considered for advanced GIST that has responded to imatinib. Several retrospective studies have suggested that adding surgical resection to imatinib at the maximum response, including partial response and stable disease, may be associated with long-term survival [112–116]. Although two randomized prospective trials conducted in Europe (NCT00956072) and China were prematurely terminated due to poor patient accrual, the study in China suggested a possible survival benefit from surgical resection in patients whose tumors were controlled by imatinib [117]. Focal progression is another scenario where a limited number of lesions display intratumoral nodules or increased size, while other lesions remain relatively stable and well-controlled [112, 118, 119]. Given the lack of definitive evidence, surgical resection is not routinely recommended for metastatic or recurrent GIST that respond to imatinib. The indications of surgical resection for advanced GIST during drug therapy should be individualized and discussed by a multidisciplinary team of experts (Table 1; see CQ4-4 in the Supplementary Material for details).

## Limitations

Several limitations should be noted. First, this is a narrative review based on the consensus of multidisciplinary experts in East Asia, not a systematic review. A formal systematic literature search with a predefined search strategy, and formal evidence grading were not performed. Second, this review did not consider country-specific regulatory approval, reimbursement status, or insurance coverage, and the implementation of drug therapies and molecular testing in clinical practice may vary across Asian countries.

## Conclusion

The Asian GIST guidelines were updated from the 2016 version based on new evidence. These guidelines can serve as consensus clinical practice tools for providing optimal care for the Asian population and facilitating shared decision-making between clinical practitioners and patients. Of note, geographic disparities, such as the availability of approved drugs and genetic testing, must be considered and need to be resolved to allow harmonization of the implementation of evidence-based treatment in Asian countries.

## Supplementary information

Below is the link to the electronic supplementary material.Supplementary file 1 (DOCX 6081 KB)
