# Supplementary material for: Updated Asian consensus guidelines for the diagnosis and management of gastrointestinal stromal tumor (2025)
Source: Gastric Cancer. 2026 Jul 15;29(5):887–903. doi: 10.1007/s10120-026-01773-4 (PMC13525072; doi:10.1007/s10120-026-01773-4)
Supplement: Supplementary file 1 [file 10120_2026_1773_MOESM1_ESM.docx]

# Online Resource 1

Clinical questions and explanatory text

# Title:

Updated Asian Consensus Guidelines for the Diagnosis and Management of Gastrointestinal Stromal Tumor (2025)

# Running title:

Asian GIST consensus guideline in 2025

# Authors name:

Hidekazu Hirano^1^, Yoichi Naito^2^, Tsuyoshi Takahashi^3^, Seiichi Hirota^4^, Kyoung-Mee Kim^5^, Min-Hee Ryu^6^, Jian Wang^7^, Hui Cao^8^, Jian Li^9^, Chien-Feng Li^10^, Chueh-Chuan Yen^11, 12^, Han-Kwang Yang^13^, Hwoon-Yong Jung^14^, Chun-Nan Yeh^15^, Hsiu-Po Wang^16^, Li-Tzong Chen^17^, Lin Shen^9^, Yoon-Koo Kang^6, 18^, Toshirou Nishida^19,20*^

# Affiliation:

1. Department of Gastrointestinal Medical Oncology, National Cancer Center Hospital, Tokyo, Japan
2. Department of General Internal Medicine/Experimental Therapeutics/Medical Oncology, National Cancer Center Hospital East, Chiba, Japan
3. Department of Gastroenterological Surgery, Osaka University Graduate School of Medicine, Osaka, Japan
4. Department of Diagnostic Pathology, School of Medicine, Hyogo Medical University, Nishinomiya, Japan
5. Department of Pathology and Translational Genomics, Samsung Medical Center, Sungkyunkwan University School of Medicine, Seoul, Republic of Korea
6. Department of Oncology, Asan Medical Center, University of Ulsan College of Medicine, Seoul, Republic of Korea
7. Department of Pathology, Fudan University Shanghai Cancer Center, Shanghai, China
8. Department of Gastrointestinal Surgery, Renji Hospital, Shanghai Jiao Tong University School of Medicine, Shanghai, China
9. State Key Laboratory of Holistic Integrative Management of Gastrointestinal Cancers, Beijing Key Laboratory of Cell & Gene Therapy for Solid Tumor, Department of GI Oncology, Peking University Cancer Hospital & Institute, Beijing, China
10. National Institute of Cancer Research, National Health Research Institutes, Tainan, Taiwan
11. Division of Clinical Research, Department of Medical Research, and Division of Medical Oncology, Center for Immuno-oncology, Department of Oncology, Taipei Veterans General Hospital, Taipei, Taiwan
12. School of Medicine, College of Medicine, and Institute of Biopharmaceutical Sciences, College of Pharmaceutical Sciences, National Yang Ming Chiao Tung University, Taipei, Taiwan
13. Department of Surgery, Seoul National University Hospital, Seoul, Republic of Korea
14. Department of Gastroenterology, Asan Medical Center, University of Ulsan College of Medicine, Seoul, Republic of Korea
15. Department of Surgery, Chang Gung Memorial Hospital, Linkou and Chang Gung University, Taipei, Taiwan
16. Department of Internal Medicine, National Taiwan University Hospital, National Taiwan University College of Medicine, Taipei, Taiwan
17. Department of Internal Medicine, Kaohsiung Medical University Hospital, and College of Medicine and Center for Cancer Research, Kaohsiung Medical University, Kaohsiung, Taiwan
18. Department of Oncology, Uijeongbu Eulji Medical Center, Eulji University, Uijeongbu-si, Republic of Korea
19. Department of Surgery, Japan Community Healthcare Organization Osaka Hospital, Osaka, Japan
20. Rare Cancer Center, National Cancer Center, Tokyo, Japan

*** Corresponding author:**

Toshirou Nishida,

Department of Surgery, Japan Community Healthcare Organization Osaka Hospital, Osaka, Japan.

E-mail: [nishida-toshirou@osaka.jcho.go.jp](mailto:nishida-toshirou@osaka.jcho.go.jp) (ORCID: 0000-0002-1542-6317)

Tel: +81-6-6441-5451

Postal address: 4-2-78, Fukushima, Fukushima Ward, Osaka, 553-0003

This supplementary material contains the clinical questions (CQs) and their explanatory texts, supplementing the descriptions presented in the main manuscript.

### CQ1-1. Are mutational analyses recommended in clinical practice for GIST?

**Recommendation**

**1-1-1:** Mutational analyses, including NGS techniques, are recommended when the diagnosis of GIST is indeterminate based on morphological and immunohistochemical findings.

**1-1-2:** Mutational analyses, including NGS techniques, are recommended for advanced or metastatic GIST as well as in the neoadjuvant or adjuvant setting.

Both formalin-fixed paraffin-embedded tissue and fresh-frozen tissue specimens are suitable for molecular analysis. When tumors show typical histology for GIST and c-KIT and/or DOG-1 immunopositivity, mutational analyses are not always required to confirm the diagnosis of GIST. However, mutational analyses, including NGS techniques, may be considered for diagnosing GIST when the tumor shows negative c-KIT/DOG-1 staining. In most instances, a c-KIT/DOG-1-negative tumor may not be a GIST. Thus, reevaluation of the tumor type using histological, immunohistochemical and mutational examinations is required. Consultation with expert pathologists for the diagnosis of GIST is also proposed. Sanger sequencing is widely available in most countries, while NGS sequencing is not widely available due to technical and/or financial constraints. However, NGS offers significant advantages, as it can simultaneously detect various gene mutations and fusion genes. For example, although rare in GIST, identification of *NTRK* fusion genes may directly lead to the use of tropomyosin receptor kinase (TRK) inhibitors, regardless of whether the tumor is classified as a GIST. Thus, NGS may be recommended in cases that are diagnostically challenging. In addition, these techniques may be useful for exploring other treatment strategies after the completion of the standard treatment strategies for GIST. Mutational analyses with NGS techniques are recommended in routine practice for patients with advanced or metastatic GIST, before the initiation of molecular targeted therapy. This is because drug sensitivity varies among mutational types, and it may be possible to predict the prognosis based on the mutational type. GISTs with some types of *PDGFRA* exon 18 mutations, especially D842V mutations, *NF1*-related GISTs, SDH-negative GISTs and *BRAF*-mutated GISTs are generally insensitive to tyrosine kinase inhibitors, including imatinib, sunitinib, regorafenib, and ripretinib.

In summary, mutational analyses including NGS techniques may be recommended for all cases of GIST or cases in which GIST is suspected. At the very least, mutational analyses should be done before starting tyrosine kinase inhibitors. Sanger sequencing and NGS sequencing should be chosen appropriately according to the situation.

### CQ2-1. Is a biopsy necessary for the diagnosis of GIST before initiating preoperative or systemic therapy?

**Recommendation**

**2-1-1:** A biopsy is recommended to confirm the diagnosis of primary GIST before initiating preoperative therapy, particularly in cases where neoadjuvant or systemic treatment is being considered.

**2-1-2:** Tissue sampling using endoscopic ultrasound-guided fine-needle aspiration (EUS-FNA) or fine-needle biopsy (EUS-FNB) is preferred for its safety and effectiveness in minimizing the risk of tumor hemorrhage or peritoneal dissemination.

**2-1-3:** Percutaneous image-guided biopsy is generally not recommended for primary localized tumors but may be acceptable for metastatic or recurrent disease, or when EUS-FNA/FNB is not feasible in locally advanced cases.

Conventional endoscopic forceps biopsy has limited diagnostic utility for GIST. In contrast, EUS-FNA and EUS-FNB are both safe and diagnostically effective, with tissue acquisition rates depending on tumor location and operator expertise [1-3]. EUS-FNB yields a larger amount of tissue, has a higher tissue acquisition rate, and offers superior diagnostic accuracy compared with EUS-FNA [4]. Alternative methods, such as mucosal incision-assisted biopsy, have also shown diagnostic accuracy and safety comparable to EUS-guided techniques [5].

While EUS-FNA or EUS-FNB is preferred due to its lower risk of tumor dissemination, percutaneous image-guided biopsy may be cautiously considered when EUS-guided sampling is not feasible—particularly in cases of metastatic or recurrent disease. Retrospective studies have suggested that, when followed by appropriate adjuvant therapy, percutaneous biopsy does not increase the risk of recurrence in patients with high-risk localized GIST [6, 7]. In light of these observations, the use of percutaneous image-guided biopsy in locally advanced but potentially resectable tumors should be individualized, taking into account the tumor location, the risk of needle tract seeding, and the availability of systemic therapy. Although not generally recommended for primary localized GIST, percutaneous biopsy may be acceptable when there is no safer diagnostic option and pathological confirmation is essential for treatment planning.

### CQ2-2. Is laparoscopic (assisted) surgery recommended for the resection of GIST?

**Recommendation**

**2-2-1:** Laparoscopic surgery is recommended for smaller GIST in favorable anatomical locations, provided that standard surgical principles—such as preserving the pseudocapsule and avoiding tumor rupture—are strictly followed.

**2-2-2:** For larger GIST, laparoscopic surgery may be considered in select cases, but its application should be individualized based on tumor size, location, and growth pattern.

Comparative studies indicate that laparoscopic surgery offers similar oncologic outcomes to open surgery, with advantages including reduced pain, minimal invasiveness, faster recovery, and improved cosmetic results [8]. These benefits, combined with its minimally invasive nature, have established laparoscopic surgery as a safe and effective option for appropriately selected patients. Laparoscopic surgery is suitable for smaller GISTs in accessible locations, such as the greater curvature or anterior stomach wall, jejunum, or ileum [9, 10]. Tumor handling should aim to minimize the risk of rupture by utilizing stapling techniques and extraction bags [9]. Retrospective studies further suggest that laparoscopic surgery may be feasible for larger GISTs, although its use for tumors > 8 cm is supported by only limited evidence and requires careful planning and multidisciplinary evaluation [10-12].

Recent advancements in laparoscopy and digestive endoscopy have also enabled laparoscopic and endoscopic cooperative surgery, further expanding the minimally invasive options for gastric GIST while achieving favorable oncologic outcomes [13, 14]. Endoscopic techniques, such as endoscopic submucosal dissection and endoscopic full-thickness resection, have evolved and are increasingly applied to small gastrointestinal (GI) submucosal tumors, including GIST. While reports suggest their efficacy and safety in managing small GIST, evidence regarding long-term oncologic outcomes is limited. Therefore, the use of endoscopic full-thickness resection for small GIST is weakly discouraged at this time due to concerns about the risk of potential tumor spillage and uncertainties in relation to its impact on long-term recurrence and survival [15-18].

### CQ2-3. Is tumor rupture associated with poor prognosis in patients with GIST?

**Recommendation**

**2-3-1:** Tumor rupture, whether occurring preoperatively or intraoperatively, is strongly associated with poor prognosis in patients with GIST, including an increased risk of recurrence.

**2-3-2:** To minimize the risk of tumor rupture, careful surgical techniques and thorough preoperative planning are essential, particularly for large or high-risk tumors.

Tumor rupture is presumably associated with micrometastatic disease. In patients with GIST, tumor rupture has been consistently associated with a significantly poor prognosis in multiple retrospective studies [19-21]. As a result, it has been incorporated into recurrence risk classifications, such as the modified NIH criteria, where it is recognized as an independent prognostic factor and defined as a high-risk feature [22]. These findings highlight the critical need for careful surgical techniques and preoperative planning to minimize the risk of tumor rupture. However, these retrospective studies have limitations, as tumor rupture was not consistently defined, potentially affecting the accuracy of prognostic assessments.

To address this limitation, a universal definition of tumor rupture has been proposed, which includes tumor fracture, spillage, blood-stained ascites, perforation at the tumor site, adjacent organ infiltration, piecemeal resection, or incisional biopsy [23, 24]. This definition has been used to retrospectively identify GIST patients at particularly high risk of recurrence. Further prospective studies are needed to clarify the prognostic impact of tumor rupture according to this definition. Additionally, a prospective observational study is warranted to evaluate the oncologic outcomes of patients experiencing tumor rupture.

### CQ2-4. Is routine postoperative follow-up useful for completely resected GIST?

**Recommendation**

**2-4-1:** Routine postoperative follow-up is weakly recommended for patients with completely resected GIST.

**2-4-2:** Follow-up schedules should be based on the risk of recurrence, time elapsed since surgery, and use of adjuvant therapy.

The benefit of routine postoperative follow-up for completely resected GIST is supported primarily by expert consensus [25-27], as no interventional studies have confirmed its impact on survival or quality of life. However, some observational studies suggest that the early detection of recurrence may improve outcomes. For example, asymptomatic detection and smaller tumor burden (e.g., ≤ 3 liver metastases or peritoneal metastases with a total tumor diameter ≤ 10 cm) have been associated with better progression-free survival (PFS) and disease-specific survival [28]. Additionally, patients with metastatic lesions of < 12 cm in size who received imatinib therapy showed a significantly longer time to progression compared to those with lesions ≥ 12 cm [29]. Although these findings suggest that the early detection of recurrence has potential benefits, they are limited by lead-time bias and classified as low-level evidence. Routine follow-up should aim to balance the early detection of recurrence with resource utilization and patient needs.

For GIST patients in high- or intermediate-risk groups, postoperative surveillance is recommended with abdominal and pelvic CT scans every 3-4 months for the first 3 years after surgery, every 6 months until the fifth year, and annually thereafter [27, 28]. For patients in low- or very low-risk groups, follow-up CT every 6 months is recommended for 5 years after surgery. Ultrasonography may replace CT once per year [27, 30]. The role of positron emission tomography (PET) in postoperative surveillance has not been established.

### CQ3-1. What is recommended after imatinib, sunitinib, and regorafenib?

**Recommendation**

**3-1-1:** Ripretinib is strongly recommended.

**3-1-2:** Pimitespib is recommended.

There are two randomized phase 3 trials evaluating the benefit of newer agents in patients with GIST previously treated with imatinib, sunitinib, and regorafenib.

Ripretinib is a novel oral inhibitor of *KIT* and *PDGFRA*. The randomized phase 3 INVICTUS study compared ripretinib versus placebo [31]. Ripretinib significantly improved PFS (median PFS: ripretinib, 6.3; placebo, 1.0 months; hazard ratio [HR], 0.15; p < 0.0001) and showed a trend toward improvement in overall survival (OS; median OS: ripretinib, 15.1 months; placebo, 6.6 months; HR, 0.36), although a formal statistical analysis was not performed based on hierarchical testing of endpoints (as objective response rate [ORR] was not significantly different). The most common adverse events were alopecia (49%), followed by myalgia (28%), nausea (26%), and fatigue (26%). Toxicities were generally mild and well tolerated. Pimitespib is an orally available heat shock protein 90 inhibitor that is currently only available in Japan. Pimitespib was evaluated against placebo in a randomized phase 3 trial [32]. Pimitespib showed a significant improvement in PFS (median PFS: pimitespib, 2.8 months; placebo, 1.4 months; HR, 0.51; p = 0.006). The median OS was significantly improved with pimitespib (median OS in the rank preserving structural failure time model adjusting for the crossover effect: pimitespib, 13.8 months; placebo, 7.6 months; HR, 0.42; p = 0.007). The most common adverse events were diarrhea (74.1%) followed by decreased appetite (31%). Treatment-emergent serious adverse events were reported in 10.6% of patients. Based on these results, the panel recommends both ripretinib and pimitespib. The panel discussed the magnitude of benefits and toxicities, resulting in a strong recommendation for ripretinib and a recommendation for pimitespib.

To date, no study has directly compared ripretinib and pimitespib, and the optimal treatment sequence or whether the combination of these agents would show superior efficacy is unknown. Currently, ripretinib is being compared with sunitinib in a randomized phase 3 trial, INSIGHT, in patients with GIST previously treated with imatinib and with a secondary mutation in *KIT* exon 17 and/or exon 18 [33]. Pimitespib in combination with imatinib after failure of imatinib is also being compared with sunitinib in a phase I study (jRCT2011210044) [34].

When these agents are not available or have failed, rechallenge with previously used treatments that were formerly effective may be considered as a therapeutic option, as exemplified by the RIGHT study, which demonstrated a benefit of imatinib rechallenge over placebo following failure of at least first-line imatinib and second-line sunitinib [35].

### CQ3-2. What is recommended for GIST without *KIT* mutations?

**Recommendation**

**3-2-1:** *PDGFRA* exon 18 mutations including D842V: avapritinib is recommended.

**3-2-2:** *NTRK1/3* fusions: entrectinib and larotrectinib are recommended.

**3-2-3:** *BRAF* V600E mutations: dabrafenib plus trametinib is recommended.

The strategy of medical therapy for GIST without *KIT* mutations differs from the standard sequence for GIST with *KIT* mutations. The most common recurrent mutations in GIST other than *KIT* mutations are *PDGFRA* mutations. The efficacy of imatinib for GIST with *PDGFRA* mutations depends on the details of the mutation. For example, D842V mutation in *PDGFRA* exon 18 is known to confer resistance to imatinib [36]. While no randomized trials have specifically focused on *PDGFRA*-mutant GIST, avapritinib demonstrated excellent activity against *PDGFRA*-mutant GIST in a phase 1 study, NAVIGATOR [37]. In this study, avapritinib demonstrated an excellent ORR (88%) in patients with *PDGFRA* D842V-mutant GIST. The most common adverse events at the phase 1 recommended dose (300 mg daily) were nausea (69%), fatigue (41%), and diarrhea (47%). Noteworthy adverse events included cognitive effects (40%) and intracranial bleeding (2%). Based on the results of NAVIGATOR, the US Food and Drug Administration (FDA) approved avapritinib for the treatment of adults with unresectable or metastatic GIST harboring *PDGFRA* exon 18 mutations, including *PDGFRA* D842V mutations.

GIST without *KIT* or *PDGFRA* mutations is sometimes referred to as wild-type GIST. Among these tumors, some types of GIST harbor *NTRK* fusion or *BRAF* mutations. For GIST with *NTRK* fusion, entrectinib and larotrectinib, the TRK inhibitors, are currently the treatment of choice. The efficacy of entrectinib in patients with solid tumors harboring oncogenic *NTRK1*, *NTRK2,* and *NTRK3* fusions was reported in the integrated analysis of three early phase studies [38]. This combined analysis included 2 patients with GIST who both responded to entrectinib [39]. Larotrectinib, another TRK inhibitor, has also shown activity against solid tumors with *NTRK* fusions. In a pooled analysis of three early phase trials [40], 4 patients with GIST were enrolled and all 4 patients responded to larotrectinib (1 complete response and 3 partial responses) [41]. Based on these results, the panel recommends entrectinib and larotrectinib for patients with GIST harboring *NTRK* fusions. *BRAF* class I V600 mutations are occasionally found in wild-type GIST. RAF inhibitors and MEK inhibitors have been reported to be active against solid tumors harboring *BRAF* class I mutations. The phase 2 ROAR study evaluated dabrafenib and trametinib in patients with rare cancers harboring *BRAF* V600E mutations [42]. Although only 1 patient with GIST was included in the study, the efficacy of dabrafenib and trametinib was reported and the FDA approved dabrafenib and trametinib for the treatment of adult and pediatric patients aged 1 year or older with unresectable or metastatic solid tumors harboring a *BRAF* V600E mutation who have progressed after prior therapy and have no satisfactory alternative treatment options. SDH-deficient GIST is one of the most common wild-type GIST. SDH deficiency can be caused either by mutations in the *SDHA*, *SDHB*, *SDHC*, or *SDHD* gene or by epigenetic mechanisms that lead to the silencing of SDHC (inactivation by promoter hypermethylation) [43]. Several targeted therapies have been evaluated in small prospective or retrospective studies. Unfortunately, the efficacy of imatinib in SDH-deficient GIST is limited [44, 45]. Sunitinib and regorafenib have shown some activity, with ORRs of approximately 20-30% [46, 47]. Other than *PDGFRA* mutations, *NTRK* fusions, and *BRAF* V600E mutations, there is little evidence to support targeted agents for wild-type GIST, and the panel makes no recommendations. The panel considers that sunitinib and regorafenib may be considered for patients with metastatic or recurrent SDH-deficient GIST. Active surveillance may be considered as a management option for wild-type GIST with an indolent clinical course.

### CQ3-3. Are higher doses of imatinib recommended?

**Recommendation**

**3-3-1:** Dose escalation of imatinib is recommended for GIST with *KIT* exon 9 mutations.

To date, no randomized trial has evaluated the benefit of dose escalation in GIST that progressed on imatinib 400 mg/day. A meta-analysis suggested that high-dose imatinib (800 mg/day) was associated with significantly higher ORR and longer PFS, but not OS, in patients with *KIT* exon 9 mutations [48]. Therefore, the panel acknowledged that a higher dose of imatinib may be considered for patients with GIST harboring *KIT* exon 9 mutations, especially for those who experience disease progression while receiving the standard dose of 400 mg per day. However, in case-control studies and systematic reviews comparing imatinib with sunitinib, imatinib dose escalation was inferior to sunitinib in terms of PFS, and there was no difference in OS [49-52]. Therefore, the use of dose escalation in patients with *KIT* exon 9 mutant GIST may be an option to be used with caution in Asian GIST patients if they are deemed likely to tolerate for more than the standard dose of imatinib. It should be noted that a retrospective study found no survival benefit with an imatinib dose of 800 mg compared with 400 mg in patients with *KIT* exon 9 mutated GIST in the adjuvant setting [53].

Monitoring of imatinib plasma levels is utilized in clinical practice as supportive information for clinical decision-making when considering dose adjustment. In this regard, dose escalation may be indicated for patients with sub-therapeutic imatinib plasma concentrations who are considered able to tolerate an increased imatinib dose.

### CQ3-4. Is starting with a lower dose of multikinase inhibitor acceptable?

**Recommendation**

**3-4-1:** Starting at a lower dose may be considered for patients who have comorbidities or who are unlikely or deemed unable to tolerate the standard dose.

To date, there have been no studies comparing the standard dose with a lower starting dose. Reducing the dose without appropriate justification may result in a reduced therapeutic effect and is not recommended. The panel considered that starting at a lower dose may be an acceptable option for patients who have comorbidities or who are unable to tolerate the standard dose. It has also been shown that the drug is not less effective when the dose is appropriately adjusted for adverse events [54], dose optimization in individual patients should be based on comorbidities and toxicities.

The usefulness of measuring blood levels of imatinib in patients with metastatic or recurrent GIST has yet to be established. With regard to the association between plasma levels of imatinib and antitumor activity, some retrospective studies have reported that imatinib plasma levels are associated with PFS [55] although there are conflicting data [56-58]. Therefore, the panel currently makes no strong recommendation and only suggests that the measurement of blood levels of imatinib may be considered under limited circumstances, such as dose escalation after imatinib dose reduction, confirmation of drug compliance, and dose reduction of imatinib [59].

### CQ4-1. What is the optimal duration of adjuvant imatinib therapy for high-risk GIST after radical resection?

**Recommendation**

**4-1-1:** Adjuvant imatinib therapy for 3 years is strongly recommended.

**4-1-2:** Adjuvant imatinib therapy for 6 years can be recommended.

**4-1-3:** Extended or lifelong adjuvant imatinib therapy is recommended for ruptured GIST.

The optimal duration of administration for patients receiving adjuvant therapy with imatinib is still under consideration. The IMADGIST study is a multicenter, open-label, randomized phase 3 trial that compared a 3-year extension of imatinib therapy versus treatment interruption in patients with high-risk GIST who had completed 3 years of adjuvant therapy with imatinib [60]. In this study, a significant improvement in disease-free survival was observed in the 3-year extension group, although the evaluation of OS was still immature. Another study is underway to investigate whether a longer duration of adjuvant imatinib therapy reduces recurrence events (NCT02413736: 5 years versus 3 years). It should be noted that the duration of adjuvant imatinib may vary across countries because of differences in national reimbursement policies.

Before the establishment of adjuvant imatinib therapy as the standard of care for high-risk GIST, most recurrences occurred in the liver or peritoneum within 2 years of surgery [26]. In the era of adjuvant imatinib therapy, the risk of recurrence remains highest within the first 1–2 years after the completion of treatment [61]. Careful follow-up is warranted after the completion of adjuvant therapy with imatinib because an increase in the recurrence rate approximately 1–2 years after the discontinuation of imatinib has been observed in previous studies [62-64].

Extended or lifelong adjuvant imatinib therapy is an option for patients with tumor rupture, as those patients are considered to have an increased risk of recurrence. While a retrospective study indicated that prolonged imatinib treatment following surgical resection was associated with favorable outcomes in patients with tumor rupture, further studies are needed to determine the optimal duration of adjuvant imatinib therapy for these patients [65].

There is no consensus regarding the optimal timing of the initiation of adjuvant imatinib therapy for localized GIST after curative surgery. In the previous pivotal studies, the interval from surgical resection to randomization or the initiation of imatinib was approximately 12 weeks [62-64, 66]. A retrospective study reported the possibility of impaired survival by delaying adjuvant imatinib therapy (> 4 months) [67]. Given the potential detrimental effect of delaying adjuvant imatinib therapy, the panel recommends initiating adjuvant therapy within approximately 12 weeks.

A genotype analysis of patients being evaluated for adjuvant imatinib therapy is preferable for identifying imatinib-insensitive GIST, including *PDGFRA* D842 V mutant GIST and wild-type GIST. Adjuvant imatinib therapy is not recommended for GIST with *PDGFRA* D842V mutations, which confer resistance to imatinib. Wild-type GIST also shows lower sensitivity to imatinib. It is unlikely to benefit from adjuvant imatinib therapy, especially in patients with syndromic GIST such as NF-1–related GIST, the Carney triad, or Carney-Stratakis syndrome [68, 69].

### CQ4-2. Is imatinib useful as neoadjuvant therapy for large GIST?

**Recommendation**

**4-2-1:** Neoadjuvant imatinib therapy is recommended for large GIST (e.g., > 10 cm in diameter), GIST for which curative resection is considered highly unlikely, and GIST located in a specific area where multivisceral resection is needed.

Neoadjuvant therapy can be considered to increase the likelihood of curability as well as the safety of surgery and organ function preservation in cases involving large GIST (e.g., > 10 cm in diameter) and GIST that is difficult to radically resect without significant morbidity. Several single-arm phase 2 trials and retrospective studies have indicated that neoadjuvant imatinib therapy results in high R0 resection rates without an increase in surgical morbidity or mortality [69-74].

It is preferable to perform mutation analyses whenever possible to avoid ineffective neoadjuvant therapy for GIST with primary resistance mutations to imatinib (e.g., *PDGFRA* D842 mutated GIST, wild type GIST). Imatinib is the only drug with evidence to support its use in the neoadjuvant setting. There is no evidence to support the use of other drugs, including tyrosine kinase inhibitors (e.g., sunitinib, regorafenib, ripretinib, pimitespib) in neoadjuvant therapy. In an expert opinion, avapritinib as neoadjuvant therapy for GIST with *PDGFRA* D842V mutation is a therapeutic option considering its high ORR in the metastatic setting. The accumulation of further evidence is necessary for evaluating the efficacy and safety of avapritinib in the neoadjuvant setting.

The optimal duration of neoadjuvant imatinib therapy has yet to be determined. Based on the time to a partial response in advanced GIST, six months of treatment is required to achieve near maximum tumor shrinkage [75, 76]. Nevertheless, further shrinkage after the 12-month time point is rare and prolonged administration may be associated with the emergence of tumor progression due to secondary resistance. Therefore, it is recommended that surgery be performed after 6–12 months of neoadjuvant imatinib therapy. Early assessment (e.g., after the first month of treatment) of the response to neoadjuvant therapy by CT and/or PET is preferable to ensure the potential of curative resection for non-responding GIST. It is unnecessary to withhold imatinib before surgery in cases without significant imatinib-related adverse events.

Considering the pathological modifications related to imatinib, the indications for adjuvant imatinib therapy should be determined based on the disease status before administering neoadjuvant therapy, as the risk classification for recurrence using surgical specimens cannot be applied. Pre-treatment clinicopathological information (e.g., tumor size, primary tumor site, mitotic index in biopsy samples, presence of tumor rupture) is useful when considering adjuvant imatinib therapy. The optimal duration for adjuvant imatinib therapy is not established after surgical resection following neoadjuvant imatinib therapy. It is important to note that recurrence-free survival is dependent on the duration of adjuvant therapy even after neoadjuvant therapy with R0 resection [74, 77]. Thus, the panel recommends that the total duration of neoadjuvant and adjuvant imatinib therapy should be at least 3 years (3–6 years) for high-risk GIST.

### CQ4-3. Is surgical resection useful as an initial treatment for metastatic or recurrent GIST?

**Recommendation**

**4-3-1:** Surgical resection as an initial treatment is not recommended for metastatic or recurrent GIST in general.

**4-3-2:** Surgical resection can be adopted as initial treatment for metastatic or recurrent GIST requiring local tumor control and for metastatic or recurrent wild-type GIST for which no effective systemic treatment is available.

Micrometastasis or circulating tumor cells may be present at the diagnosis of metastatic or recurrent disease. Therefore, surgical resection as an initial treatment is not generally recommended for metastatic or recurrent GIST. In a retrospective study, initial debulking surgery prior to imatinib did not improve the prognosis of patients with advanced GIST [78]. In a prospective study for GIST with liver oligometastasis, all patients had hepatic recurrence after surgical resection [79]. There are no established subgroups of patients with advanced GIST for whom upfront surgical resection has proven beneficial. Given the limited evidence on the benefits of initial surgical resection in patients with metastasis, further studies are necessary to investigate the clinical value of this approach, particularly in patients with single- or oligo-metastatic recurrence with a long disease-free interval. Upfront surgical resection may be considered in specific situations where local tumor control is crucial (e.g., hemorrhage, invasion to GI tract, obstruction of GI tract, risk of tumor rupture by drug response) and in patients who have wild-type GIST for which no effective systemic treatment is available.

### CQ4-4. Is surgical resection useful for metastatic or recurrent GIST that respond to drug therapy?

**Recommendation**

**4-4-1:** Surgical resection is not routinely recommended for metastatic or recurrent GIST that respond to imatinib.

**4-4-2:** Given the lack of definitive evidence, the indications of surgical resection for advanced GIST during drug therapy should be individualized and discussed by a multidisciplinary team of experts.

Systemic treatment rarely eradicates all tumor cells, resulting in a low pathological complete response rate due to the existence and/or emergence of drug-resistant tumor clones. Drug-resistant tumor clones are thought to be present before treatment and/or develop over time after treatment. Surgical resection may play a role in eliminating or delaying the development of tumors that have drug resistance.

Surgical resection can be considered for advanced GIST that have responded to imatinib. Although the optimal duration of imatinib therapy is unclear, the panel recommends 6–12 months of imatinib therapy before surgical resection to achieve the maximum response. Imatinib can be administered up to the day before surgical resection and restarted when patients can eat. Imatinib should be continued after surgical resection. Several retrospective studies have suggested that adding surgical resection to imatinib at the maximum response, including partial response and stable disease, may be associated with long-term survival [80-84]. Two randomized prospective trials were conducted in Europe (NCT00956072) and China to evaluate the survival benefit of surgical resection in patients with advanced GIST that had responded to imatinib [85]. However, these trials were prematurely terminated due to poor patient accrual. Nevertheless, the study in China suggested a possible survival benefit from surgical resection in patients whose tumors were controlled by imatinib [85]. The 2-year PFS rate was 88.4% in the surgical resection group and 57.7% in the non-surgical resection group (p = 0.089). The median OS was not reached in the surgical resection group, whereas it was 49 months in the non-surgical resection group (p = 0.024). Focal progression is a scenario where a limited number of lesions display intratumoral nodules or increased size, along with increased PET uptake, while other lesions remain relatively stable and well-controlled. The potential benefits of surgical resection have also been proposed in patients with focal progression in retrospective studies [80, 86, 87]. In patients with multifocal progression, characterized by the simultaneous progression of multiple lesions, surgical resection is not expected to be effective. Therefore, surgical resection is generally not recommended for patients with multifocal progression except for symptom palliation.

There is limited evidence to support the recommendation of surgical resection during systemic treatment with sunitinib, regorafenib, ripretinib, avapritinib, or pimitespib. Surgical resection might be beneficial for carefully selected patients (e.g., patients with a limited number of metastases who achieve long-term stable disease after the systemic treatment).

# References

1.	Akahoshi K, Oya M, Koga T, Shiratsuchi Y. Current clinical management of gastrointestinal stromal tumor. World J Gastroenterol. 2018;24:2806-17.

2.	Zhang XC, Li QL, Yu YF, Yao LQ, Xu MD, Zhang YQ, et al. Diagnostic efficacy of endoscopic ultrasound-guided needle sampling for upper gastrointestinal subepithelial lesions: a meta-analysis. Surg Endosc. 2016;30:2431-41.

3.	Cazacu IM, Singh BS, Luzuriaga Chavez AA, Koduru P, Ejaz S, Weston BR, et al. EUS and EUS-guided FNA/core biopsies in the evaluation of subepithelial lesions of the lower gastrointestinal tract: 10-year experience. Endosc Ultrasound. 2020;9:329-36.

4.	Facciorusso A, Sunny SP, Del Prete V, Antonino M, Muscatiello N. Comparison between fine-needle biopsy and fine-needle aspiration for EUS-guided sampling of subepithelial lesions: a meta-analysis. Gastrointest Endosc. 2020;91:14-22.e2.

5.	Osoegawa T, Minoda Y, Ihara E, Komori K, Aso A, Goto A, et al. Mucosal incision-assisted biopsy versus endoscopic ultrasound-guided fine-needle aspiration with a rapid on-site evaluation for gastric subepithelial lesions: A randomized cross-over study. Dig Endosc. 2019;31:413-21.

6.	Yeh CH, Pan KT, Chu SY, Chen CM, Hsu MY, Hung CF, et al. Safety and efficacy of image-guided percutaneous biopsies in the diagnosis of gastrointestinal stromal tumors. Clin Imaging. 2012;36:19-23.

7.	Eriksson M, Reichardt P, Sundby Hall K, Schütte J, Cameron S, Hohenberger P, et al. Needle biopsy through the abdominal wall for the diagnosis of gastrointestinal stromal tumour - Does it increase the risk for tumour cell seeding and recurrence? Eur J Cancer. 2016;59:128-33.

8.	Nishimura J, Nakajima K, Omori T, Takahashi T, Nishitani A, Ito T, et al. Surgical strategy for gastric gastrointestinal stromal tumors: laparoscopic vs. open resection. Surg Endosc. 2007;21:875-8.

9.	Novitsky YW, Kercher KW, Sing RF, Heniford BT. Long-term outcomes of laparoscopic resection of gastric gastrointestinal stromal tumors. Ann Surg. 2006;243:738-45; discussion 45-7.

10.	Takahashi T, Nakajima K, Miyazaki Y, Miyazaki Y, Kurokawa Y, Yamasaki M, et al. Surgical strategy for the gastric gastrointestinal stromal tumors (GISTs) larger than 5 cm: laparoscopic surgery is feasible, safe, and oncologically acceptable. Surg Laparosc Endosc Percutan Tech. 2015;25:114-8.

11.	Lian X, Feng F, Guo M, Cai L, Liu Z, Liu S, et al. Meta-analysis comparing laparoscopic versus open resection for gastric gastrointestinal stromal tumors larger than 5 cm. BMC Cancer. 2017;17:760.

12.	Cui JX, Gao YH, Xi HQ, Cai AZ, Zhang KC, Li JY, et al. Comparison between laparoscopic and open surgery for large gastrointestinal stromal tumors: A meta-analysis. World J Gastrointest Oncol. 2018;10:48-55.

13.	Hiki N, Nunobe S, Matsuda T, Hirasawa T, Yamamoto Y, Yamaguchi T. Laparoscopic endoscopic cooperative surgery. Dig Endosc. 2015;27:197-204.

14.	Hiki N, Nunobe S. Laparoscopic endoscopic cooperative surgery (LECS) for the gastrointestinal tract: Updated indications. Ann Gastroenterol Surg. 2019;3:239-46.

15.	Meng Y, Li W, Han L, Zhang Q, Gong W, Cai J, et al. Long-term outcomes of endoscopic submucosal dissection versus laparoscopic resection for gastric stromal tumors less than 2 cm. J Gastroenterol Hepatol. 2017;32:1693-7.

16.	Ko EJ, Bang BW, Kwon KS, Shin YW, Kim HK. Endoscopic Enucleation Is Effective and Relatively Safe in Small Gastric Subepithelial Tumors Originating from Muscularis Propria. Dig Dis Sci. 2019;64:524-31.

17.	Li B, Chen T, Qi ZP, Yao LQ, Xu MD, Shi Q, et al. Efficacy and safety of endoscopic resection for small submucosal tumors originating from the muscularis propria layer in the gastric fundus. Surg Endosc. 2019;33:2553-61.

18.	Zhai YQ, Chai NL, Li HK, Lu ZS, Feng XX, Zhang WG, et al. Endoscopic submucosal excavation and endoscopic full-thickness resection for gastric schwannoma: five-year experience from a large tertiary center in China. Surg Endosc. 2020;34:4943-9.

19.	Takahashi T, Nakajima K, Nishitani A, Souma Y, Hirota S, Sawa Y, et al. An enhanced risk-group stratification system for more practical prognostication of clinically malignant gastrointestinal stromal tumors. Int J Clin Oncol. 2007;12:369-74.

20.	Rutkowski P, Bylina E, Wozniak A, Nowecki ZI, Osuch C, Matlok M, et al. Validation of the Joensuu risk criteria for primary resectable gastrointestinal stromal tumour - the impact of tumour rupture on patient outcomes. Eur J Surg Oncol. 2011;37:890-6.

21.	Hølmebakk T, Hompland I, Bjerkehagen B, Stoldt S, Bruland Ø S, Hall KS, et al. Recurrence-Free Survival After Resection of Gastric Gastrointestinal Stromal Tumors Classified According to a Strict Definition of Tumor Rupture: A Population-Based Study. Ann Surg Oncol. 2018;25:1133-9.

22.	Joensuu H, Vehtari A, Riihimaki J, Nishida T, Steigen SE, Brabec P, et al. Risk of recurrence of gastrointestinal stromal tumour after surgery: an analysis of pooled population-based cohorts. Lancet Oncol. 2012;13:265-74.

23.	Nishida T, Hølmebakk T, Raut CP, Rutkowski P. Defining Tumor Rupture in Gastrointestinal Stromal Tumor. Ann Surg Oncol. 2019;26:1669-75.

24.	Gotohda N, Nishida T, Sato S, Ozaka M, Nakahara Y, Komatsu Y, et al. Re-appraisal of the universal definition of tumor rupture among patients with high-risk gastrointestinal stromal tumors. Ann Gastroenterol Surg. 2023;7:1021-31.

25.	Cavnar MJ, Seier K, Curtin C, Balachandran VP, Coit DG, Yoon SS, et al. Outcome of 1000 Patients With Gastrointestinal Stromal Tumor (GIST) Treated by Surgery in the Pre- and Post-imatinib Eras. Ann Surg. 2021;273:128-38.

26.	Wada N, Takahashi T, Kurokawa Y, Nakajima K, Masuzawa T, Nakatsuka R, et al. Appropriate Follow-Up Strategies for Gastrointestinal Stromal Tumor Patients Based on the Analysis of Recurrent Interval and Patterns. Digestion. 2017;95:115-21.

27.	Joensuu H, Martin-Broto J, Nishida T, Reichardt P, Schöffski P, Maki RG. Follow-up strategies for patients with gastrointestinal stromal tumour treated with or without adjuvant imatinib after surgery. Eur J Cancer. 2015;51:1611-7.

28.	D'Ambrosio L, Palesandro E, Boccone P, Tolomeo F, Miano S, Galizia D, et al. Impact of a risk-based follow-up in patients affected by gastrointestinal stromal tumour. Eur J Cancer. 2017;78:122-32.

29.	Van Glabbeke M, Verweij J, Casali PG, Le Cesne A, Hohenberger P, Ray-Coquard I, et al. Initial and late resistance to imatinib in advanced gastrointestinal stromal tumors are predicted by different prognostic factors: a European Organisation for Research and Treatment of Cancer-Italian Sarcoma Group-Australasian Gastrointestinal Trials Group study. J Clin Oncol. 2005;23:5795-804.

30.	Joensuu H, Reichardt P, Eriksson M, Sundby Hall K, Vehtari A. Gastrointestinal stromal tumor: a method for optimizing the timing of CT scans in the follow-up of cancer patients. Radiology. 2014;271:96-103.

31.	Blay JY, Serrano C, Heinrich MC, Zalcberg J, Bauer S, Gelderblom H, et al. Ripretinib in patients with advanced gastrointestinal stromal tumours (INVICTUS): a double-blind, randomised, placebo-controlled, phase 3 trial. Lancet Oncol. 2020;21:923-34.

32.	Kurokawa Y, Honma Y, Sawaki A, Naito Y, Iwagami S, Komatsu Y, et al. Pimitespib in patients with advanced gastrointestinal stromal tumor (CHAPTER-GIST-301): a randomized, double-blind, placebo-controlled phase III trial. Ann Oncol. 2022;33:959-67.

33.	George S, Blay JY, Chi P, Jones RL, Serrano C, Somaiah N, et al. The INSIGHT study: a randomized, Phase III study of ripretinib versus sunitinib for advanced gastrointestinal stromal tumor with KIT exon 11 + 17/18 mutations. Future Oncol. 2024;20:1973-82.

34.	Naito Y, Komatsu Y, Kurokawa Y, Hirano H, Nishida T, Doi T. A phase I study of pimitespib combined with imatinib in patients with imatinib-refractory gastrointestinal stromal tumor. Ann Oncol. 2023;34(Suppl):S1031–S1032.

35.	Kang YK, Ryu MH, Yoo C, Ryoo BY, Kim HJ, Lee JJ, et al. Resumption of imatinib to control metastatic or unresectable gastrointestinal stromal tumours after failure of imatinib and sunitinib (RIGHT): a randomised, placebo-controlled, phase 3 trial. Lancet Oncol. 2013;14:1175-82.

36.	Corless CL, Schroeder A, Griffith D, Town A, McGreevey L, Harrell P, et al. PDGFRA mutations in gastrointestinal stromal tumors: frequency, spectrum and in vitro sensitivity to imatinib. J Clin Oncol. 2005;23:5357-64.

37.	Heinrich MC, Jones RL, von Mehren M, Schöffski P, Serrano C, Kang YK, et al. Avapritinib in advanced PDGFRA D842V-mutant gastrointestinal stromal tumour (NAVIGATOR): a multicentre, open-label, phase 1 trial. Lancet Oncol. 2020;21:935-46.

38.	Doebele RC, Drilon A, Paz-Ares L, Siena S, Shaw AT, Farago AF, et al. Entrectinib in patients with advanced or metastatic NTRK fusion-positive solid tumours: integrated analysis of three phase 1-2 trials. Lancet Oncol. 2020;21:271-82.

39.	Loong HHF, Tinoco G, Garrido-Laguna I, Goto K, Steuer C, Liu SV, et al. Updated efficacy and safety of entrectinib in patients with NTRK fusion-positive sarcomas. Connective Tissue Oncology Society (CTOS) Annual Meeting; 2022.

40.	Hong DS, DuBois SG, Kummar S, Farago AF, Albert CM, Rohrberg KS, et al. Larotrectinib in patients with TRK fusion-positive solid tumours: a pooled analysis of three phase 1/2 clinical trials. Lancet Oncol. 2020;21:531-40.

41.	Demetri GD, Tan DS, Bielack S, Orbach D, DuBois S, Federman NC, et al. Larotrectinib efficacy and safety in patients with TRK fusion sarcomas. Connective Tissue Oncology Society (CTOS) Annual Meeting; 2019.

42.	Subbiah V, Kreitman RJ, Wainberg ZA, Gazzah A, Lassen U, Stein A, et al. Dabrafenib plus trametinib in BRAFV600E-mutated rare cancers: the phase 2 ROAR trial. Nat Med. 2023;29:1103-12.

43.	Nannini M, Rizzo A, Indio V, Schipani A, Astolfi A, Pantaleo MA. Targeted therapy in SDH-deficient GIST. Ther Adv Med Oncol. 2021;13:17588359211023278.

44.	Heinrich MC, Rankin C, Blanke CD, Demetri GD, Borden EC, Ryan CW, et al. Correlation of Long-term Results of Imatinib in Advanced Gastrointestinal Stromal Tumors With Next-Generation Sequencing Results: Analysis of Phase 3 SWOG Intergroup Trial S0033. JAMA Oncol. 2017;3:944-52.

45.	Boikos SA, Pappo AS, Killian JK, LaQuaglia MP, Weldon CB, George S, et al. Molecular Subtypes of KIT/PDGFRA Wild-Type Gastrointestinal Stromal Tumors: A Report From the National Institutes of Health Gastrointestinal Stromal Tumor Clinic. JAMA Oncol. 2016;2:922-8.

46.	Ben-Ami E, Barysauskas CM, von Mehren M, Heinrich MC, Corless CL, Butrynski JE, et al. Long-term follow-up results of the multicenter phase II trial of regorafenib in patients with metastatic and/or unresectable GI stromal tumor after failure of standard tyrosine kinase inhibitor therapy. Ann Oncol. 2016;27:1794-9.

47.	Liu W, Zeng X, Wu X, He J, Gao J, Shuai X, et al. Clinicopathologic study of succinate-dehydrogenase-deficient gastrointestinal stromal tumors: A single-institutional experience in China. Medicine (Baltimore). 2017;96:e7668.

48.	Gastrointestinal Stromal Tumor Meta-Analysis Group (MetaGIST). Comparison of two doses of imatinib for the treatment of unresectable or metastatic gastrointestinal stromal tumors: a meta-analysis of 1,640 patients. J Clin Oncol. 2010;28:1247-53.

49.	Hislop J, Mowatt G, Sharma P, Fraser C, Elders A, Jenkinson D, et al. Systematic review of escalated imatinib doses compared with sunitinib or best supportive care, for the treatment of people with unresectable/metastatic gastrointestinal stromal tumours whose disease has progressed on the standard imatinib dose. J Gastrointest Cancer. 2012;43:168-76.

50.	Hislop J, Quayyum Z, Elders A, Fraser C, Jenkinson D, Mowatt G, et al. Clinical effectiveness and cost-effectiveness of imatinib dose escalation for the treatment of unresectable and/or metastatic gastrointestinal stromal tumours that have progressed on treatment at a dose of 400 mg/day: a systematic review and economic evaluation. Health Technol Assess. 2011;15:1-178.

51.	Vincenzi B, Nannini M, Fumagalli E, Bronte G, Frezza AM, De Lisi D, et al. Imatinib dose escalation versus sunitinib as a second line treatment in KIT exon 11 mutated GIST: a retrospective analysis. Oncotarget. 2016;7:69412-9.

52.	Dong Z, Gao J, Gong J, Li J, Li Y, Shen L, et al. Clinical benefit of sunitinib in gastrointestinal stromal tumors with different exon 11 mutation genotypes. Future Oncol. 2017;13:2035-43.

53.	Vincenzi B, Napolitano A, Fiocco M, Mir O, Rutkowski P, Blay JY, et al. Adjuvant Imatinib in Patients with GIST Harboring Exon 9 KIT Mutations: Results from a Multi-institutional European Retrospective Study. Clin Cancer Res. 2022;28:1672-9.

54.	Reichardt P, Kang YK, Rutkowski P, Schuette J, Rosen LS, Seddon B, et al. Clinical outcomes of patients with advanced gastrointestinal stromal tumors: safety and efficacy in a worldwide treatment-use trial of sunitinib. Cancer. 2015;121:1405-13.

55.	Demetri GD, Wang Y, Wehrle E, Racine A, Nikolova Z, Blanke CD, et al. Imatinib plasma levels are correlated with clinical benefit in patients with unresectable/metastatic gastrointestinal stromal tumors. J Clin Oncol. 2009;27:3141-7.

56.	Yoo C, Ryu MH, Ryoo BY, Beck MY, Kang YK. Efficacy, safety, and pharmacokinetics of imatinib dose escalation to 800 mg/day in patients with advanced gastrointestinal stromal tumors. Invest New Drugs. 2013;31:1367-74.

57.	Eechoute K, Fransson MN, Reyners AK, de Jong FA, Sparreboom A, van der Graaf WT, et al. A long-term prospective population pharmacokinetic study on imatinib plasma concentrations in GIST patients. Clin Cancer Res. 2012;18:5780-7.

58.	Koo DH, Ryu MH, Ryoo BY, Beck MY, Na YS, Shin JG, et al. Association of ABCG2 polymorphism with clinical efficacy of imatinib in patients with gastrointestinal stromal tumor. Cancer Chemother Pharmacol. 2015;75:173-82.

59.	Hirota S, Tateishi U, Nakamoto Y, Yamamoto H, Sakurai S, Kikuchi H, et al. English version of Japanese Clinical Practice Guidelines 2022 for gastrointestinal stromal tumor (GIST) issued by the Japan Society of Clinical Oncology. Int J Clin Oncol. 2024;29:647-80.

60.	Blay JY, Schiffler C, Bouché O, Brahmi M, Duffaud F, Toulmonde M, et al. A randomized study of 6 versus 3 years of adjuvant imatinib in patients with localized GIST at high risk of relapse. Ann Oncol. 2024;Sep 4:S0923-7534(24)03924-3.

61.	Ushimaru Y, Takahashi T, Nakajima K, Teranishi R, Nishida T, Hirota S, et al. Real-world data on the efficacy and safety of adjuvant chemotherapy in Japanese patients with a high-risk of gastrointestinal stromal tumor recurrence. Int J Clin Oncol. 2022;27:921-9.

62.	Dematteo RP, Ballman KV, Antonescu CR, Maki RG, Pisters PW, Demetri GD, et al. Adjuvant imatinib mesylate after resection of localised, primary gastrointestinal stromal tumour: a randomised, double-blind, placebo-controlled trial. Lancet. 2009;373:1097-104.

63.	Joensuu H, Eriksson M, Sundby Hall K, Hartmann JT, Pink D, Schütte J, et al. One vs Three Years of Adjuvant Imatinib for Operable Gastrointestinal Stromal Tumor. JAMA. 2012;307:1265.

64.	Casali PG, Le Cesne A, Poveda Velasco A, Kotasek D, Rutkowski P, Hohenberger P, et al. Time to Definitive Failure to the First Tyrosine Kinase Inhibitor in Localized GI Stromal Tumors Treated With Imatinib As an Adjuvant: A European Organisation for Research and Treatment of Cancer Soft Tissue and Bone Sarcoma Group Intergroup Randomized Trial in Collaboration With the Australasian Gastro-Intestinal Trials Group, UNICANCER, French Sarcoma Group, Italian Sarcoma Group, and Spanish Group for Research on Sarcomas. J Clin Oncol. 2015;33:4276-83.

65.	Kang S, Ryu MH, Bang YH, Kim HD, Lee HE, Kang YK. Adjuvant Imatinib Treatment for 5 Years versus 3 Years in Patients with Ruptured Localized Gastrointestinal Stromal Tumor: A Retrospective Analysis. Cancer Res Treat. 2022;54:1167-74.

66.	Raut CP, Espat NJ, Maki RG, Araujo DM, Trent J, Williams TF, et al. Efficacy and Tolerability of 5-Year Adjuvant Imatinib Treatment for Patients With Resected Intermediate- or High-Risk Primary Gastrointestinal Stromal Tumor. JAMA Oncol. 2018;4:e184060.

67.	Qianyi W, Mei X, Rui Z, Yong W, Yutao W, Xiaoding S, et al. Delayed adjuvant imatinib in patients with high risk of recurrence of gastrointestinal stromal tumor after radical surgery: a retrospective cohort study. J Cancer Res Clin Oncol. 2022;148:1493-500.

68.	Janeway KA, Kim SY, Lodish M, Nose V, Rustin P, Gaal J, et al. Defects in succinate dehydrogenase in gastrointestinal stromal tumors lacking KIT and PDGFRA mutations. Proc Natl Acad Sci U S A. 2011;108:314-8.

69.	Wang D, Zhang Q, Blanke CD, Demetri GD, Heinrich MC, Watson JC, et al. Phase II Trial of Neoadjuvant/adjuvant Imatinib Mesylate for Advanced Primary and Metastatic/recurrent Operable Gastrointestinal Stromal Tumors: Long-term Follow-up Results of Radiation Therapy Oncology Group 0132. Ann Surg Oncol. 2012;19:1074-80.

70.	Eisenberg BL, Harris J, Blanke CD, Demetri GD, Heinrich MC, Watson JC, et al. Phase II trial of neoadjuvant/adjuvant imatinib mesylate (IM) for advanced primary and metastatic/recurrent operable gastrointestinal stromal tumor (GIST): Early results of RTOG 0132/ACRIN 6665. J Surg Oncol. 2009;99:42-7.

71.	Kurokawa Y, Yang H-K, Cho H, Ryu M-H, Masuzawa T, Park SR, et al. Phase II study of neoadjuvant imatinib in large gastrointestinal stromal tumours of the stomach. Br J Cancer. 2017;117:25-32.

72.	Tielen R, Verhoef C, Van Coevorden F, Gelderblom H, Sleijfer S, Hartgrink HH, et al. Surgical treatment of locally advanced, non-metastatic, gastrointestinal stromal tumours after treatment with imatinib. Eur J Surg Oncol. 2013;39:150-5.

73.	Mcauliffe JC, Hunt KK, Lazar AJF, Choi H, Qiao W, Thall P, et al. A Randomized, Phase II Study of Preoperative plus Postoperative Imatinib in GIST: Evidence of Rapid Radiographic Response and Temporal Induction of Tumor Cell Apoptosis. Ann Surg Oncol. 2009;16:910-9.

74.	Rutkowski P, Gronchi A, Hohenberger P, Bonvalot S, Schöffski P, Bauer S, et al. Neoadjuvant Imatinib in Locally Advanced Gastrointestinal Stromal Tumors (GIST): The EORTC STBSG Experience. Ann Surg Oncol. 2013;20:2937-43.

75.	Blanke CD, Demetri GD, von Mehren M, Heinrich MC, Eisenberg B, Fletcher JA, et al. Long-term results from a randomized phase II trial of standard- versus higher-dose imatinib mesylate for patients with unresectable or metastatic gastrointestinal stromal tumors expressing KIT. J Clin Oncol. 2008;26:620-5.

76.	Nishida T, Shirao K, Sawaki A, Koseki M, Okamura T, Ohtsu A, et al. Efficacy and safety profile of imatinib mesylate (ST1571) in Japanese patients with advanced gastrointestinal stromal tumors: a phase II study (STI571B1202). Int J Clin Oncol. 2008;13:244-51.

77.	Kong SH, Kurokawa Y, Yook JH, Cho H, Kwon OK, Masuzawa T, et al. Long-term outcomes of a phase II study of neoadjuvant imatinib in large gastrointestinal stromal tumors of the stomach. Gastric Cancer. 2023;26:775-87.

78.	An HJ, Ryu M-H, Ryoo B-Y, Sohn BS, Kim K-H, Oh ST, et al. The Effects of Surgical Cytoreduction Prior to Imatinib Therapy on the Prognosis of Patients with Advanced GIST. Ann Surg Oncol. 2013;20:4212-8.

79.	Kanda T, Masuzawa T, Hirai T, Ikawa O, Takagane A, Hata Y, et al. Surgery and imatinib therapy for liver oligometastasis of GIST: a study of Japanese Study Group on GIST. Jpn J Clin Oncol. 2017;47:369-72.

80.	Raut CP, Posner M, Desai J, Morgan JA, George S, Zahrieh D, et al. Surgical Management of Advanced Gastrointestinal Stromal Tumors After Treatment With Targeted Systemic Therapy Using Kinase Inhibitors. J Clin Oncol. 2006;24:2325-31.

81.	Sym SJ, Ryu MH, Lee JL, Chang HM, Kim TW, Kim HC, et al. Surgical intervention following imatinib treatment in patients with advanced gastrointestinal stromal tumors (GISTs). J Surg Oncol. 2008;98:27-33.

82.	Fairweather M, Balachandran VP, Li GZ, Bertagnolli MM, Antonescu C, Tap W, et al. Cytoreductive Surgery for Metastatic Gastrointestinal Stromal Tumors Treated With Tyrosine Kinase Inhibitors. Ann Surg. 2018;268:296-302.

83.	Park SJ, Ryu M-H, Ryoo B-Y, Park YS, Sohn BS, Kim HJ, et al. The Role of Surgical Resection Following Imatinib Treatment in Patients with Recurrent or Metastatic Gastrointestinal Stromal Tumors: Results of Propensity Score Analyses. Ann Surg Oncol. 2014;21:4211-7.

84.	Bauer S, Rutkowski P, Hohenberger P, Miceli R, Fumagalli E, Siedlecki JA, et al. Long-term follow-up of patients with GIST undergoing metastasectomy in the era of imatinib -- analysis of prognostic factors (EORTC-STBSG collaborative study). Eur J Surg Oncol. 2014;40:412-9.

85.	Du CY, Zhou Y, Song C, Wang YP, Jie ZG, He YL, et al. Is there a role of surgery in patients with recurrent or metastatic gastrointestinal stromal tumours responding to imatinib: a prospective randomised trial in China. Eur J Cancer. 2014;50:1772-8.

86.	Yeh CN, Chen TW, Tseng JH, Liu YY, Wang SY, Tsai CY, et al. Surgical management in metastatic gastrointestinal stromal tumor (GIST) patients after imatinib mesylate treatment. J Surg Oncol. 2010;102:599-603.

87.	Cho H, Ryu MH, Lee Y, Park YS, Kim KH, Kim JH, et al. Role of Resection Following Focal Progression with Standard Doses of Imatinib in Patients with Advanced Gastrointestinal Stromal Tumors: Results of Propensity Score Analyses. Oncologist. 2019;24:e1443-e9.
